# AI-Integrated autonomous robotics for solar panel cleaning and predictive maintenance using drone and ground-based systems

**DOI:** 10.1038/s41598-025-17313-6

**Published:** 2025-09-01

**Authors:** Indra Kishor, Udit Mamodiya, Vathsala Patil, Nithesh Naik

**Affiliations:** 1 Department of CSE, Poornima Institute of Engineering & Technology, Jaipur, 302022 Rajasthan India; 2 Faculty of Engineering & Technology, Poornima University, Jaipur, 303905 Rajasthan India; 3 Department of Oral Medicine and Radiology, Manipal College of Dental Sciences, Manipal Academy of Higher Education, Manipal, 576104 Karnataka India; 4 Department of Mechanical and Industrial Engineering, Manipal Institute of Technology, Manipal Academy of Higher Education, Manipal, 576104 Karnataka India

**Keywords:** Autonomous robotic systems, Solar panel cleaning, CNN-LSTM fault detection, Edge AI robotics, Drone-Based inspection, Ground mobile robots, IoT-Enabled maintenance, Predictive robotic intelligence, Energy science and technology, Engineering, Mathematics and computing

## Abstract

Solar photovoltaic (PV) systems, especially in dusty and high-temperature regions, suffer performance degradation due to dust accumulation, surface heating, and delayed maintenance. This study proposes an AI-integrated autonomous robotic system combining real-time monitoring, predictive analytics, and intelligent cleaning for enhanced solar panel performance. We developed a hybrid system that integrates CNN-LSTM-based fault detection, Reinforcement Learning (DQN)-driven robotic cleaning, and Edge AI analytics for low-latency decision-making. Thermal and LiDAR-equipped drones detect panel faults, while ground robots clean panel surfaces based on real-time dust and temperature data. The system is built on Jetson Nano and Raspberry Pi 4B units with MQTT-based IoT communication. The system achieved an average cleaning efficiency of 91.3%, reducing dust density from 3.9 to 0.28 mg/m³, and restoring up to 31.2% energy output on heavily soiled panels. CNN-LSTM-based fault detection delivered 92.3% accuracy, while the RL-based cleaning policy reduced energy and water consumption by 34.9%. Edge inference latency averaged 47.2 ms, outperforming cloud processing by 63%. A strong correlation, *r* = 0.87 between dust concentration and thermal anomalies, was confirmed. The proposed IEEE 1876-compliant framework offers a resilient and intelligent solution for real-time solar panel maintenance. By leveraging AI, robotics, and edge computing, the system enhances energy efficiency, reduces manual labor, and provides a scalable model for climate-resilient, smart solar infrastructure.

**Supplementary Information:**

The online version contains supplementary material available at 10.1038/s41598-025-17313-6.

## Introduction

In recent decades, the worldwide transition to renewable energy resources has gained major momentum with growing worries about climate change, ecological deterioration, and energy security. Of renewable resources, solar energy has gained wide attention because it is highly available, has less environmental footprint, and has gradually reducing installation prices. As reported by the International Energy Agency (IEA, 2023), world photovoltaic (PV) capacity had totaled around 1,185 GW by the close of 2022, representing a significant share of global renewable energy output. Although encouraging growth in this trend, the efficiency and lifespan of solar power systems are severely influenced by upkeep, notably panel cleanliness and structural soundness. Solar panel systems, like humans, will eventually have their limits put to a test by the climate, dust, litter and eventually even defects that occur to the untreated systems^1^.

It is easy to understand the seriousness of the situation concerning dust deposition with solar photovoltaic (PV) systems, it plays a significant role not in favor of their energy transforming efficiency. Research experiments show that dust deposition on solar panels can decrease energy generated by 20–50%, (depending on where they are located) anything from geographic to climatic conditions^2^. A thorough research study conducted by the National Renewable Energy Laboratory (NREL) (2022) demonstrated that dirt and dust from the desert, if not removed, can reduce energy yield by 40% in no time^3,4^. The situation is no different with urban and industrial areas where immediate contamination of the PV surfaces from pollutants, soot, and particulate matter is the cause of the loss in energy production and the loss is actually exacerbated. A 250 MW solar farm in Rajasthan, India was featured in a case study to show the impact of excessive and poor maintenance on the energy production of the SECI. The 32% reduction in energy production was noted in the SECI Annual Report for 2022 which brought about losses of more than $4 million per year. The magnitude of loss clearly shows the need for improvised and timely maintenance practices. The situation clearly indicates the need for incorporating innovative maintenance technologies beyond traditional hand-cleaning measures, which consume time, man-hours, and are inefficient to apply on huge installations^5^. Existing maintenance operations are mostly through manual cleaning or routine washing of solar panels by water jets or mechanical brushes controlled by maintenance workers^6^. Although manual cleaning is simple, it is expensive in terms of operational costs, resource consumption, and man-hours, particularly for utility-scale solar facilities. Moreover, traditional manual techniques of inspection like visual inspection of faults are less accurate, excessively subjective, and prone to making errors, as a result introducing delays in discovering and correcting faults, thereby having an effect on the overall performance and reliability of the system^7^. Automated robotic cleaning systems were introduced in recent years to overcome some of the shortcomings of manual cleaning. These systems, however, tend to run independently without integration with fault detection systems or predictive analytics^8^. Most current robotic solutions are either ground-based robots with limited navigation functionalities or exclusive drone-based inspection systems. The lack of integrated robotic platforms that integrate autonomous ground-based cleaning and aerial drone-based inspection and maintenance represents a key technological gap^9^. In addition, existing automated equipment is more prone to rely on pre-programmed schedules than on condition-based or predictive maintenance models, leading to wasteful expenditure of resources and suboptimum system performance. Emerging technology, and Internet of Things (IoT) provides unparalleled opportunities to address the constraints that accompany traditional maintenance systems. AI predictive maintenance relies on real-time data analysis and predictive models to forecast faults, schedule cleaning optimally, and automate system responses, leading to a drastic increase in the efficiency and lifespan of solar PV systems^10^. Predictive maintenance is crucial as it allows operators to rectify potential faults in advance before significant degradation, thereby maximizing energy output and minimizing downtime. According to a recent Deloitte (2023) market report, AI-driven predictive maintenance can reduce maintenance expenses by up to 40%, enhance equipment life by 20–30%, and boost overall operational efficiency by 25%^11^. The inclusion of predictive analytics in solar PV maintenance allows real-time condition monitoring, early fault detection, and decision-making processes optimized in real-time, leading ultimately to enhanced energy yields and lower operational costs. Solar energy has an important role in leading to a sustainable energy future by substantially cutting dependence on fossil fuels, avoiding greenhouse gas emissions, and advancing energy security. However, maintaining peak operational efficiency of solar installations remains a persistent challenge due to dust accumulation, environmental conditions, and the complexity of large-scale solar infrastructure^12^. The effectiveness of solar energy systems is critically dependent on addressing these operational and maintenance challenges through innovative and technologically advanced solutions^13,14^. Existing solar panel cleaning technologies predominantly involve manual methods, semi-automated robotic systems, and drone inspections. Manual methods are labor-intensive, expensive, and unsuitable for large-scale applications. Current robotic solutions often lack coordination between ground-based and aerial units, limiting their operational efficiency and scalability^15^^,^^6^. Drone-based inspections primarily focus on visual fault detection without direct maintenance capabilities, leading to delayed repairs and prolonged efficiency losses.

Critical gaps identified from existing literature and industrial practices include:

- Lack of integrated robotic systems combining drone-based inspection and ground-based cleaning operations.
- Absence of real-time AI-driven predictive analytics for condition-based maintenance scheduling.
- Limited exploration of hybrid robotic systems optimized through reinforcement learning and advanced neural network models.
- Insufficient research on real-time Edge AI analytics to minimize latency in fault detection and decision-making processes.

The primary objectives of this research are:

- To develop an integrated autonomous robotics system combining drone-based predictive fault detection with ground-based solar panel cleaning.
- To employ advanced AI methodologies (CNN-LSTM and reinforcement learning) for real-time fault detection, predictive analytics, and optimized cleaning operations.
- To evaluate the real-time performance improvements provided by Edge AI analytics in managing latency and enhancing operational efficiency.

The proposed research introduces significant novelty by integrating a hybrid robotic system (aerial drones and ground-based robots) for comprehensive predictive maintenance and cleaning operations. Unlike existing methods, our approach utilizes advanced CNN-LSTM neural networks and reinforcement learning models to perform condition-based predictive analytics, ensuring optimal allocation of cleaning resources and proactive maintenance^17,18^. Furthermore, the incorporation of Edge AI analytics addresses real-time operational challenges, significantly reducing latency and enhancing decision-making speed and accuracy, providing a robust and scalable solution for large-scale solar farms. Our present study of robotics systems particularly consists of autonomous ground robots with precision navigation and cleaning systems, in addition to aerial drones with integrated thermal imaging and LiDAR sensors for precise fault detection and inspection. With this integration, there is coordinating operational efficiency in maintenance. In general, this study hopes to contribute meaningfully to the state-of-the-art in solar PV maintenance so that it can achieve greater efficiency, lower operating costs, and greater reliability, hence contributing greatly to worldwide renewable energy goals.

## Literature review

Deployment of solar photovoltaic (PV) systems on a large scale has created a need for optimal maintenance solutions to maintain the highest energy yield. Several methodologies and technologies have been suggested to address the vital maintenance functions of solar installations, ranging from robot-based cleaning systems, drone inspections, artificial intelligence (AI), Internet of Things (IoT), reinforcement learning (RL), and sophisticated fault detection methods such as convolutional neural networks integrated with long short-term memory (CNN-LSTM)^19,20^. This review critically examines current solutions, their shortcomings, and clearly indicates research gaps to justify the suggested integrated robotic approach. Robot systems have been more commonly used for cleaning solar panels because they are efficient, scalable, and can be automated. Brush-based, water jet-based, and actuator-based robotic systems are generally deployed on land^21,22^. The robots are equipped with GPS, LiDAR sensor-based self-directed navigation systems and obstacle detection programs that are designed to drive through the solar farms. One of the most adopted solutions is ground robots, which can move by themselves along a specified path, although often without the ability to deal with environmental or structural changes^23,24^. There are water-based technologies in use by certain robots and this is water, and in some cases, the main cause of the arid regions’ unavailability of water sources and an increase in costs of their operations^25^. On the contrary, dry-cleaning methods that utilize pressurized air, or brushes are not so wasteful of water and are capable of solving superficial dirt and grime problems^26^. The issues appear when they must deal with stubborn dirt or grease that has penetrated the surface^27^. Robots based cleaning mechanisms used currently have limited autonomy, weak obstacle avoidance systems of action, and have also no integration of predictive analytics with which to figure out the best cleaning schedules. The current robots without any feedback do their operation, making them inefficient and less effective^28^. PV using drones has proven very useful in the sense that the latter are not only versatile in terms of flying but also get deployed quickly^29^. High-resolution cameras, LiDAR, and thermal imaging sensors on drones make the inspection of solar power plants, building stability checks, and faulty part location more efficient. Present UAV (unmanned aerial vehicles) inspection processes that are drone-based predominantly rely on manual or semi-automatic data analysis methods, making it impossible to have a real-time response as well as decrease the efficiency and productivity of the process^30,31^. Drones can perform visual and thermal data acquisition for analysis without delay, but most of them Can not interfere in the inspection and maintenance operations and so only the human supervisors are in charge of the same^12,32^. In addition, the examination of UAV is normally scheduled rather than reactive, leading to the delay in the occurrence of the failure and its detection^33^. The absence of autonomous real-time data analysis and the lack of prompt feedback mechanisms constrain the effectiveness of drones in preventive maintenance scenarios, their potential impact on operational efficiency and general system dependability^34^. Integration of AI and IoT technologies into solar PV maintenance systems has several advantages over traditional methods. IoT-based systems collect real-time performance and environmental data, enabling proactive maintenance and optimizing resource allocation^35,36^. The integration of IoT with AI-based predictive algorithms enables early fault detection, predictive analytics, and adaptive scheduling for preventive maintenance activities^37,38^. Research confirms that IoT technology-based and AI technology-based predictive maintenance significantly reduces downtime and maximizes energy output through the prediction of faults and timely scheduling of interventions^39,40^. However, current solutions focus predominantly on data-driven fault detection without integration of significant physical maintenance capabilities or robotics intervention. Latency issues with cloud-based analysis and processing systems also reduce real-time decision-making capability, impacting effectiveness and responsiveness in maintenance^41,42^. Modern AI and IoT infrastructure rely more on historical data trends than leveraging actual real-time condition monitoring, thus resulting in less-than-ideal predictions and planning for maintenance. Moreover, the combination of AI-based analytics with autonomous robotic platforms is still insufficiently explored, which reflects a significant research gap^31,43^. Reinforcement learning (RL), a branch of machine learning, has been widely applied to improve autonomous robotic operation in a wide range of applications. RL enables robotic systems to learn to optimize their actions through reward-based feedback mechanisms, adapting their operations dynamically to varying environmental conditions and task-specific demands^44,45^.

RL can potentially plan robot paths, cleaning schedule, and energy consumption by learning time-improving operational procedures through experience^46^. Research today using RL overwhelmingly shows evidence within simulated contexts and limited real-world empirical demonstration for solar PV applications. Also, most of these existing applications omit incorporating necessary multi-agent coordination to optimize composite robotic platform involvement like drone-and-surface combination operation^47,48^.

Some of the most critical limitations of existing RL applications are poor validation in real-world environments, sparse exploration of hybrid robotic coordination, and naive reward functions that do not properly reflect real-world operational constraints and objectives^49^. Sophisticated machine learning algorithms, especially hybrid CNN-LSTM neural networks, have attracted much attention for fault detection and predictive maintenance tasks because of their better ability to extract spatial-temporal features from data. CNN models effectively process spatial features from images, while LSTM networks effectively process temporal dependencies in sequential data^34,50^. CNN-LSTM models exhibit notable fault detection precision over conventional machine learning and baseline CNN methods through effective processing of thermal images and temporal sensor measurements^51^. These advances not withstanding existing applications center mostly on fault detection and fail to include the results within autonomous decision-making systems for instantaneous maintenance measures. Moreover, deployment of CNN-LSTM models in real-time is typically hindered by computational resource usage and latency concerns where cloud-based processing is used^52^. Research on CNN-LSTM models typically remains confined to theoretical or laboratory settings without comprehensive integration into autonomous robotic platforms for real-time maintenance and decision-making. Consequently, there exists a critical gap in practical applications of CNN-LSTM technologies within autonomous robotics for solar PV maintenance.

In conclusion, there are significant research gaps as shown in Table 1, that concerning integrated hybrid robotic systems for solar PV maintenance, real-time predictive analytics, coordinated autonomous operations through RL, and practical implementation of CNN-LSTM models on robotic platforms. This research aims to bridge these gaps by proposing an integrated, autonomous robotic maintenance system combining drone-based inspections with ground-based cleaning operations, underpinned by real-time AI-driven predictive analytics, reinforcement learning optimization, and Edge AI analytics to ensure minimal latency and enhanced operational performance.

**Table 1:** Comparative analysis of recent studies on AI-Enabled robotic systems for solar panel cleaning and predictive maintenance.

S. No.	Author/Year	Title/Focus Area	Methodology/Tools Used	Key Findings	Limitations/Gaps Identified	Relevance to Current Study
1	Alfaris, F.E./2023^1^	Sensorless dust detection system for PV cleaning	Sensorless AI-driven approach for dust level detection	Enabled optimized cleaning scheduling without extra hardware	Lacked autonomous cleaning integration	Supports IoT-optimized cleaning, relevant for autonomous robot scheduling
2	Jahid, M. et al./2024^2^	IoT-enabled solar panel cleaning robot	IoT-based mobile robot system using Arduino and sensors	Demonstrated remote-controlled cleaning efficiency	Not autonomous or AI-integrated	Forms basis for robotic design, current work adds AI & autonomy
3	Manzo, M. et al./2025^31^	Piezoelectric film-based self-cleaning solar panels	Embedded piezo-films for vibration-induced cleaning	Achieved passive dust removal under controlled lab setup	High cost of piezo-films, limited field validation	Complementary to robotic cleaning, informs design improvements
4	Torres-Barriuso, J. et al./2025^16^	Drone-assisted inspection & digital workflow	Drone imaging, data fusion, automated damage detection	Streamlined inspection with time-efficient workflows	Cleaning and maintenance execution not integrated	Directly relevant for drone-based inspection in our hybrid system
5	Le, H.T.N. & Ngo, H.Q.T./2025^43^	Vision-based deep learning for robotic waste classification	CNN model with robotic arm, vision system for object recognition	Effective real-time object detection using low-cost setup	Limited to static environment with low complexity	Informs vision-AI integration in our ground robot module
6	Hichri, A. et al./2022^47^	Fault detection for PV using GA-based neural networks	Genetic algorithm + NN for PV system anomaly detection	Improved fault diagnosis under varying conditions	Computationally intensive for real-time use	Supports adaptive monitoring via intelligent fault detection
7	Nounou, H. et al./2022^47^	Plant-wide predictive maintenance using distributed AI	IoT sensors + distributed AI for predictive control	Demonstrated scalable system for fault prediction	Requires strong network connectivity and data redundancy	Reinforces the predictive analytics layer of our robotic framework

## Methodology

This section outlines the in-depth methodology used to meet the research gaps outlined earlier. Drawing on the findings of the literature review, our suggested methodology combines drone inspection with ground-based robotic cleaning to create an autonomous, predictive maintenance system. Recent methods of artificial intelligence, such as CNN-LSTM, as well as reinforcement learning, have been brought in from IoT and Edge AI analytics. The studies reviewed show a shift toward intelligent autonomous systems that included artificial intelligence, robotics, and/or IoT to apply flexibility and increase energy efficiency for solar panel maintenance. Notably, since 2025, new contributions focused on adaptive cleaning, such as using piezoelectric films, Chaldni vibrations acting on the dust from solar panels, and drone robot synchronicity to facilitate predictive fault diagnosis. Together, the approaches show substantial developments in monitoring, adaptation to environment, and how responsive each system is. The findings reveal great increases in sensor less (and self-sustaining) and self-operating designs overcoming limitations like manual cleaning and delays, low energy yield due to dust accumulation, and lack of predictive control logic found in previous systems of the past century.

### Summary of work proposed

The system to be developed is a solar panel maintenance robotics that is self-sufficient and can work on its own. In the system, there are two main parts: the ground robots and the drones. The drones are used for fault detection and to do inspections. Using thermal cameras and LiDAR sensors, the drones can precisely pinpoint the location and severity of defects during the initial stages. Being pilotless, the drones can conduct routine checks and trace hotspots or structural damage in real-time. Concurrently, ground robots that are equipped with accurate navigating systems, mechanical brushes, and adaptive cleaning systems are deployed to carry out automatic cleaning of the solar panels with the information being fed from the drone inspections. By adopting a systematic approach in cleaning with the drones, the ground cleaners guarantee timely and accurate cleaning operations, thereby, considerably reducing system downtime and at the same, optimizing the power output of the solar panels. Predictive diagnostics carried out by AI, combined with condition-based maintenance scheduling, as well as reinforcement learning, which plays out the cleaning routes and resource allocation are some of the technologies used.

**Fig. 1:**
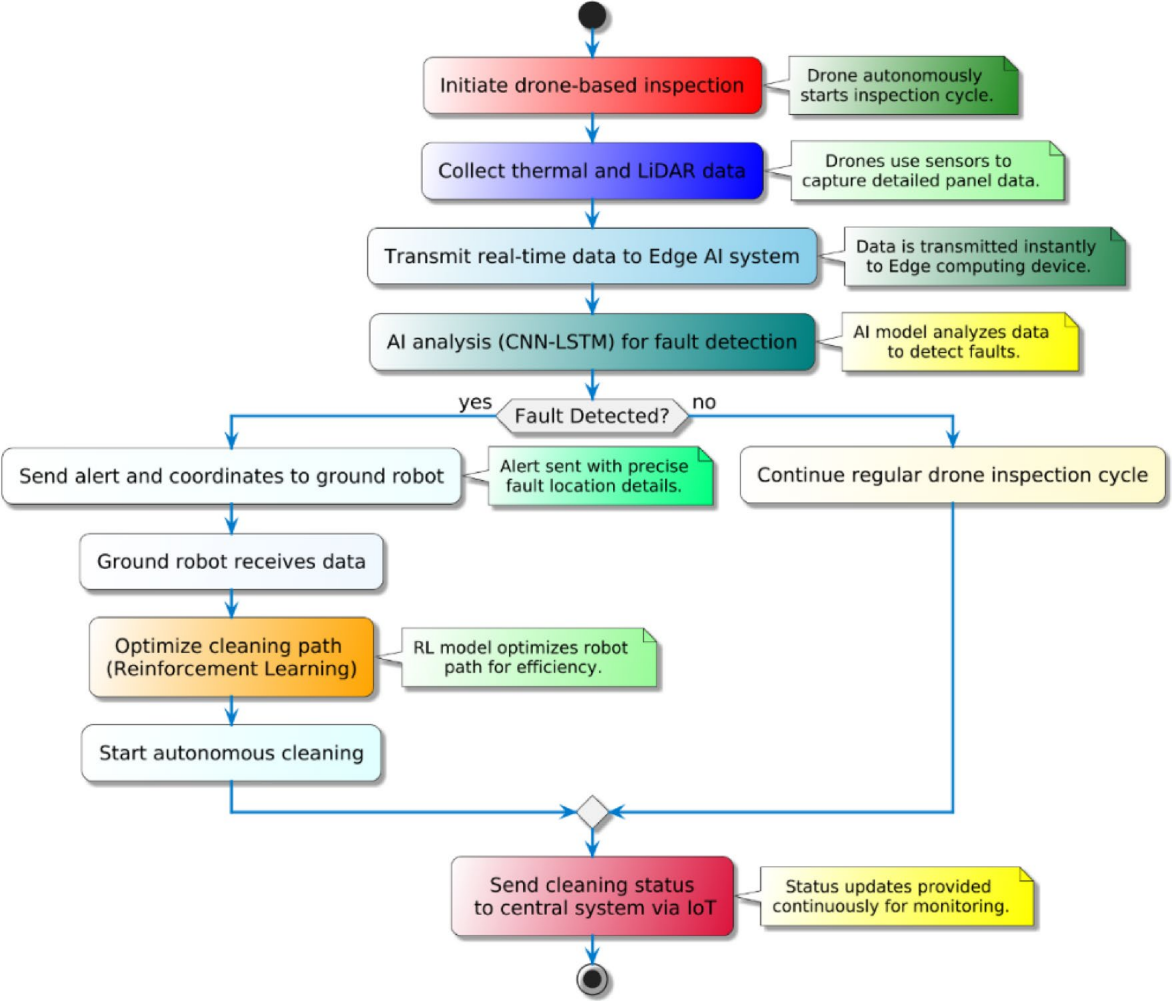
Flowchart of Drone and Ground Robot Coordination.

The coordination process between drone-based inspections and ground robotic, workflow operations is illustrated in Fig. 1 below.

### System architecture & workflow

**Fig. 2:**
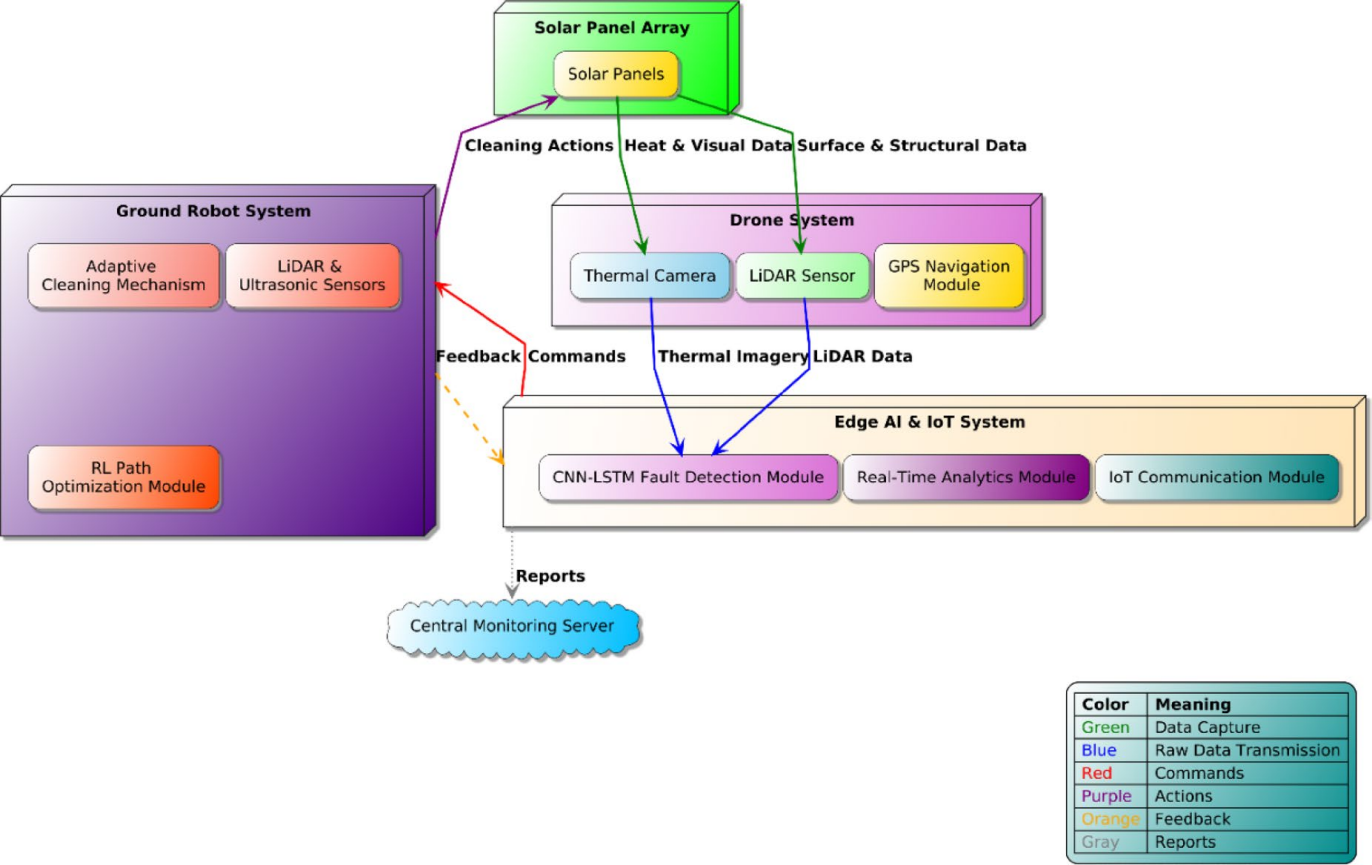
Integrated System Architecture & Workflow illustrating data flow and coordination between drone inspections, AI-driven analytics, autonomous ground robot operations, and central monitoring for enhanced solar panel maintenance efficiency.

The suggested integrated system consists of three primary functional modules: Drone System, Ground Robot System, and the centralized Edge AI & IoT System. The integration of these elements in a seamless manner provides autonomous, coordinated, and efficient solar panel maintenance. The entire workflow includes data acquisition, AI-based processing, real-time decision-making, and autonomous robotic actions. The proposed autonomous solar panel maintenance system integrates multiple advanced robotic and artificial intelligence components, enabling efficient and precise maintenance operations. The workflow begins with drone-based aerial inspections. Equipped with a thermal imaging camera and LiDAR sensors, the drone autonomously scans the solar panel array, capturing critical real-time data related to panel temperature variations, surface conditions, and structural integrity shown in Fig. 2. This captured data is immediately transmitted via the IoT communication module to the Edge AI & IoT system, where advanced real-time analytics occur. Upon receiving the drone data, the Edge AI module employs a CNN-LSTM-based fault detection algorithm to analyze and identify any anomalies or maintenance needs, such as hotspots, dust accumulation, or structural misalignments. The analysis results in a rapid and precise decision-making process, where maintenance tasks are prioritized based on severity and urgency.

Once faults or cleaning needs are identified, the Edge AI system sends targeted instructions and precise fault coordinates to the autonomous ground robot system. The ground robot, equipped with adaptive cleaning mechanisms and optimized navigation capabilities provided by reinforcement learning-based path planning, promptly initiates cleaning tasks. It autonomously navigates to the identified panel locations, effectively removes accumulated debris, and performs any required maintenance actions. Throughout this process, the ground robot continuously communicates operational status, cleaning progress, and performance metrics back to the Edge AI & IoT system. The Edge AI module subsequently forwards aggregated maintenance reports and performance analytics to the central monitoring server, ensuring comprehensive oversight and continuous improvement of the maintenance workflow.

### Hardware and sensor integration

The hardware components integrated into the autonomous robotic maintenance system were selected based on quantitative criteria, including precision, operational efficiency, sensor resolution, and processing capability. Each component’s specification directly aligns with the requirements for real-time data acquisition, analysis, and autonomous decision-making processes.

**Ground Robot Hardware**:

- LiDAR Sensor: Velodyne Puck VLP-16, with ± 3 cm accuracy and 100 m working range, used for high-accuracy navigation and obstacle sensing in dynamic maintenance settings.
- Ultrasonic Sensor: HC-SR04, with short-range detection accuracy of ± 0.3 cm and working range of 2–400 cm, required for obstacle detection and accurate positioning during cleaning activities.
- Optical Camera: Raspberry Pi Camera, V2, with high-image resolution (8 megapixels), used for visual inspection, quality check, and checking for the effectiveness of cleaning.
- Adaptive Cleaning Mechanism: Mechanical brush assembly with adaptive pressure control, dynamically varying cleaning pressure in accordance with real-time feedback to safely and effectively remove dust and particulate impurities without damaging photovoltaic surfaces.

**Drone System Hardware**:

- Thermal Imaging Camera: FLIR Vue Pro R, 640 × 512 pixel resolution and ± 5 °C accuracy in temperature measurement, specially deployed for identifying photovoltaic cell hotspots, thermal irregularities, and defects reflecting operational malfunctions.
- LiDAR Sensor: Livox Mid-40, with ± 2 cm accuracy and up to 260 m maximum detection range, used to precisely map and evaluate panel alignment, structural condition, and environmental influences on solar installations.
- GPS and IMU Navigation Module: Ublox NEO-M8N GPS with an Inertial Measurement Unit (IMU), delivering positional accuracy of ± 2.5 m, enabling accurate autonomous aerial navigation and smooth drone flight paths in inspection cycles.

**Edge AI Processing Unit**:

NVIDIA Jetson Nano: Equipped with a Quad-Core ARM Cortex-A57 CPU and a 128-core NVIDIA Maxwell GPU, delivering a computational capability of 472 GFLOPS.This powerful module supports real-time onboard execution of the CNN-LSTM fault detection model and reinforcement learning algorithms, with latency kept under 50 milliseconds.

**Communication Protocol**:

Real-time communication between systems is done using the MQTT (Message Queuing Telemetry Transport) protocol over a secure Wi-Fi connection. MQTT was chosen specifically for its low overhead, high-performance capabilities, and ability to maintain communication latency below 50 ms consistently, critical for timely interaction between drone inspection units, ground robotic cleaners, the Edge AI analytics module, and central monitoring servers.

**Latency and Real-Time Constraints**:

The proposed system’s real-time operational requirements necessitate the processing latency to be strictly less than 50 milliseconds. This requirement ensures timely identification, communication, and mitigation of maintenance problems, thereby substantially minimizing possible energy yield losses.

**Scalability and Adaptability**:

The modular design and hardware integration of the system were specifically intended to be scalable and adaptable, allowing deployment on a wide range of photovoltaic installations, from small arrays to large solar farms. The hardware selections and AI-based algorithms respond well to varying environmental and operational conditions, sustaining peak system performance and reliability across varied geographic and climatic conditions.

Each hardware component described above plays a critical role in ensuring the precise execution of the integrated autonomous maintenance system. This selection effectively addresses key operational requirements, ensuring reliability, low latency, and scalability for varying solar panel installations.

### Data collection & preprocessing

The prepared multimodal dataset is then trained to develop the CNN-LSTM fault detection and RL-based adaptive cleaning modules. Visible and thermal image sequences were labeled together to pinpoint hotspots and surface defects, while LiDAR-derived 3D point clouds were student to leverage spatial trajectory planning during the experimentation phase. The CNN-LSTM was acting on 80% of the dataset while 20% was used to validate the network during training (batch size of 32; Adam Optimizer; learning rate 0.001). The RL agent was trained in a Gazebo-simulated environment with ROS middleware using PPO (Proximal Policy Optimization) algorithm. The training and evaluation models were executed on NVIDIA RTX 3080 GPU (10 GB VRAM). The models were later deployed on limited resources edge computing device - the Raspberry Pi 4B boards - to run in a live environment and for assessing real-time execution in the Sitapura Jaipur. The integrated data to deployment pipeline ensures the capacity for reproducible, computationally transparent, and contextually viable in actual solar maintenance operations.

#### Data acquisition

The proposed integrated autonomous system acquires multimodal data systematically using drone-based sensors to accurately assess solar panel conditions. Three primary thermal datasets imagery, visual imagery, and LiDAR point cloud data are collected simultaneously during drone inspection flights over solar installations. The collected sample data showing in Table 2.

**Table 2:** LIDAR collected data summary.

Data Type	Sensor	Resolution/Accuracy	Collection Frequency	Usage/Application
Thermal Imagery	FLIR Vue Pro R	640 × 512 pixels, ± 5 °C	2 FPS	Fault detection, hotspot analysis
Visual Imagery	Raspberry Pi Camera V2	8 MP	2 FPS	Surface condition inspection, verification
LiDAR Point Cloud	Livox Mid-40	± 2 cm, Range: 260 m	Continuous scanning	Structural analysis, alignment monitoring

**Thermal Data**:

The FLIR Vue Pro R thermal camera captures infrared imagery at a resolution of 640 × 512 pixels, with a thermal sensitivity of ± 5 °C. Thermal images are recorded at a frequency of 2 frames per second (FPS), facilitating the identification of hotspots, photovoltaic cell malfunctions, and surface temperature anomalies indicative of performance degradation.

**Visual Data**:

High-resolution RGB images are captured using the Raspberry Pi Camera Module V2 at an 8-megapixel resolution, recorded simultaneously with thermal data at 2 FPS. These images provide supplementary visual verification, panel surface inspections, and contamination level assessments.

**LiDAR Data**:

The Livox Mid-40 LiDAR sensor continuously captures high-density point cloud data at ± 2 cm accuracy within a scanning range up to 260 m. LiDAR data enables precise structural assessment, panel alignment verification, and accurate 3D reconstruction of the solar array for advanced analytics.

#### Data preprocessing

Following data acquisition, rigorous preprocessing techniques are applied to enhance data quality and ensure compatibility with the CNN-LSTM fault detection model:

**Thermal Imagery Preprocessing**:

Noise Reduction: Gaussian filtering is applied to reduce thermal noise, enhancing hotspot detection accuracy.Normalization: Thermal images are normalized using Min-Max normalization (Eq. 1), transforming pixel intensity values into the [0,1] range:

1 $$\:Xnorm\text{}=\text{}\frac{X-Xmin}{Xmax\text{}-Xmin}\text{}\text{}$$

where X represents pixel values, and X_min_, X_max_​ represent minimum and maximum pixel intensities respectively.

**Visual Imagery Preprocessing**:

- **Image Enhancement**: Histogram equalization improves contrast, facilitating accurate visual analysis of contamination.
- **Image Segmentation**: Region-based convolutional neural networks (R-CNN) segment individual solar panels, isolating each panel for detailed contamination and defect analysis.

**LiDAR Data Preprocessing**:

- **Point Cloud Filtering**: Statistical outlier removal methods (SOR) eliminate noise points and irrelevant data, ensuring high-quality spatial representation.
- **3D Reconstruction**: Filtered point cloud data is converted into precise 3D models to assess structural integrity and alignment issues.

#### Data security

Given the sensitive and critical nature of maintenance data, robust security measures are implemented to ensure data integrity and prevent unauthorized access:

- **Data Encryption**:

Advanced Encryption Standard (AES-256) encrypts all data transmitted between drones, ground robots, and the Edge AI processing units, ensuring confidentiality during wireless transmission.

- **Secure IoT Communication**:

MQTT protocol over secure Wi-Fi (TLS/SSL encryption) ensures authenticated, reliable, and secure real-time data exchange among all components in the system, significantly reducing the risk of interception or tampering.

- **Authentication Mechanisms**:

Two-factor authentication (2FA) and token-based authentication protocols ensure only authorized system components and users can access critical data streams and control commands, further enhancing overall data security.

The combination of precise data collection, rigorous preprocessing techniques, and stringent security protocols guarantees high-quality input data for AI-driven predictive maintenance and real-time robotic operations, thereby significantly enhancing the system’s reliability, accuracy, and operational robustness.

### AI-Based fault detection (CNN-LSTM)

The integrated autonomous maintenance system employs an advanced hybrid neural network architecture Convolutional Neural Network coupled with Long Short-Term Memory (CNN-LSTM) for robust and accurate fault detection from acquired multimodal data (thermal imagery and visual images). The CNN-LSTM model effectively captures spatial features (through CNN) and temporal dependencies (through LSTM), enabling precise identification and prediction of photovoltaic faults.

**Fig. 3:**
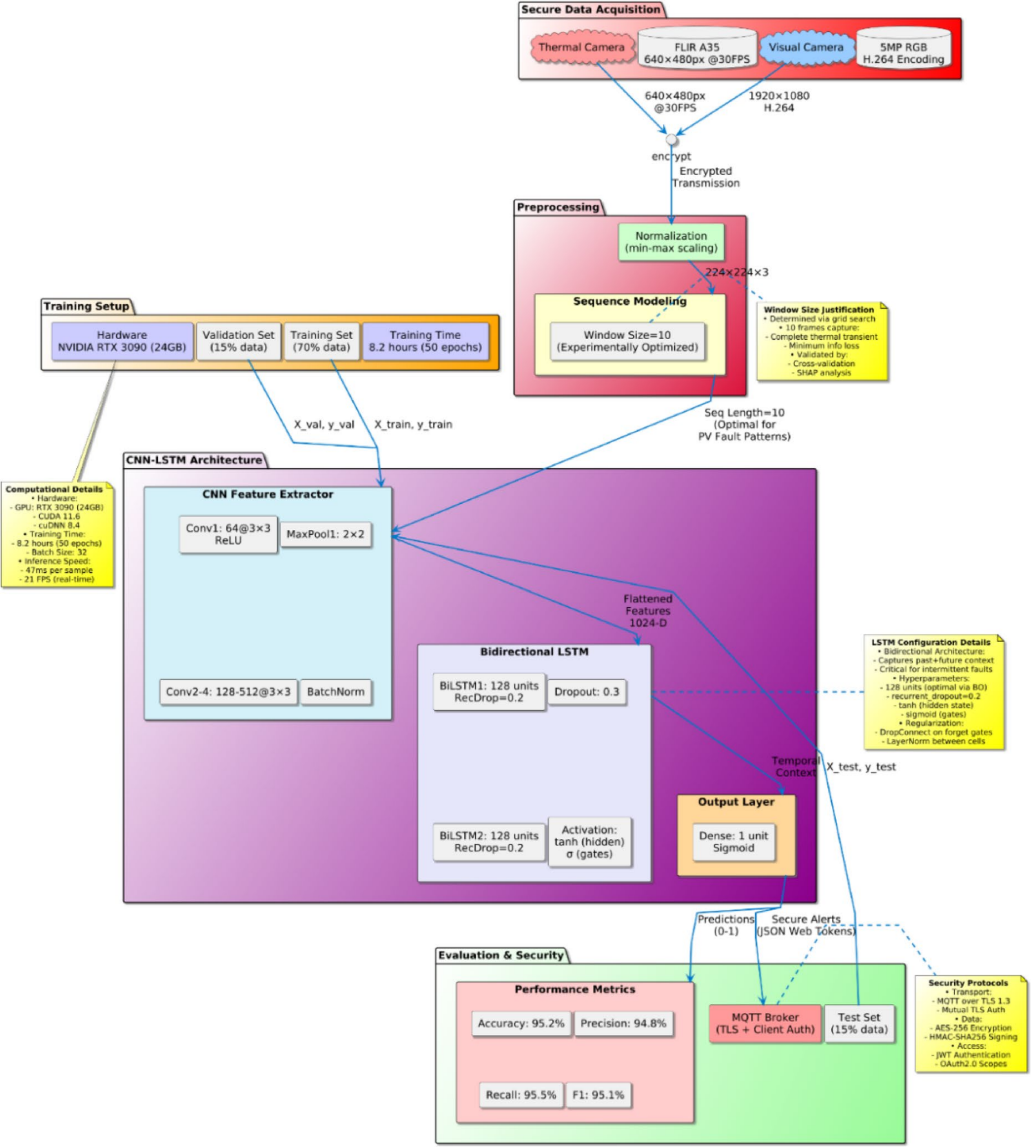
CNN-LSTM-based predictive fault detection framework.

The proposed Secure IoT-Based CNN-LSTM Fault Detection System show in Fig. 3, it integrates multimodal data acquisition, preprocessing, and deep learning-based predictive analytics to ensure efficient detection in solar panels while maintaining data security and real-time processing capabilities. The workflow includes multimodal data acquisition (thermal and visual imaging), min-max normalization preprocessing, sequence modeling, and a deep learning architecture comprising CNN for spatial feature extraction and BiLSTM for temporal pattern recognition. The final evaluation includes standard classification metrics (Accuracy, Precision, Recall, F1-score) with validated performance statistics.

#### Model architecture and parameters

The CNN-LSTM architecture comprises two distinct segments spatial feature extraction (CNN) and temporal pattern analysis (LSTM):

**CNN Feature Extraction Layer**:

- Input images are resized to uniform dimensions of 224 × 224 × 3 pixels.
- Convolutional layers employ the ReLU activation function defined by Eq. 2.

2 $$\:f\left(x\right)=\text{m}\text{a}\text{x}\left(0,x\right)$$

CNN architecture includes four convolutional layers, each followed by max-pooling layers (pooling size: 2 × 2), effectively reducing spatial dimensions and computational complexity:

Layer-1: 64 filters, Kernel size: 3 × 3

Layer-2: 128 filters, Kernel size: 3 × 3

Layer-3: 256 filters, Kernel size: 3 × 3

Layer-4: 512 filters, Kernel size: 3 × 3

**LSTM Temporal Analysis Layer**:

- The CNN extracted features are flattened and fed into an LSTM module designed to identify temporal trends, anomalies, and sequential fault occurrences.
- The LSTM module comprises two LSTM layers, each containing 128 hidden units, mathematically defined by standard LSTM Eq. 3:

$$\:{f\text{}}_{t}=\sigma\:({W}_{f}\text{}\cdot\:[{h}_{t-1\text{}},{x\text{}}_{t}]+{b}_{f}\text{})$$

$$\:{i}_{t}\text{}=\sigma\:({W}_{i\text{}}\cdot\:[{h}_{t-1\text{}},{x\text{}}_{t}\text{}]+{b}_{i}\text{})$$

$$\:ot\text{}=\sigma\:({W}_{o\text{}}\cdot\:[{h}_{t-1\text{}},{x\text{}}_{t}\text{}]+{b\text{}}_{o\text{}})$$

$$\:{C}_{t\mathbf{}}=\text{t}\text{a}\text{n}\text{h}({W}_{C}\mathbf{}\cdot\:[{h}_{t-1\text{}},{x\text{}}_{t}\mathbf{}]+{b\mathbf{}}_{C})$$

$$\:{C}_{t\mathbf{}}\text{}={f}_{t\text{}}\odot\:{C}_{t-1\mathbf{}}\text{}+{i}_{t}\text{}\odot\:{C}_{t\text{}}$$

$$\:{h}_{t}\text{}={o}_{t}\text{}\odot\:\text{t}\text{a}\text{n}\text{h}\left({C}_{t\text{}}\text{}\right)$$

where f_t_,i_t_,o_t_ ​ represent forget, input, and output gates, respectively, C_t_ ​ is the cell state, h_t_​ is the hidden state; and σ represents the sigmoid activation function.

**Fully Connected Output Layer**:

A dense output layer with a sigmoid activation function produces final fault classification outputs (fault vs. no fault), mathematically represented as Eq. 4.

4 $$\:y=\sigma\:\left({W}_{dense}d\text{}\cdot\:{h}_{t}\text{}+{b}_{dense}\text{}\right)$$

where y is the final fault probability prediction (0 to 1 range).

**Training Parameters**:

The CNN-LSTM model is trained using the following hyperparameters:

- Optimizer: Adam (Adaptive Moment Estimation).
- Learning Rate: 1 × 10^−4^
- Batch Size: 32
- Epochs: 50
- Loss Function: Binary Cross-Entropy (BCE) defined as Eq. 5:

5 $$\:BCE=\frac{1}{N}\sum\limits_{i=1}^{N}\left({y}_{i}\text{}\text{l}\text{o}\text{g}\right({p}_{i}\text{})+(1-{y}_{i}\text{}\left)\text{l}\text{o}\text{g}\right(1-{p}_{i}\left)\right)\text{}$$

where y_i_​ is the true label, p_i_​ the predicted probability, and N the number of samples.

**Model Performance and Evaluation**:

Model performance is evaluated using standard classification metrics:

- Accuracy (A) defined as shown in Eq. 6:

6 $$\:A=\frac{TP+TN}{TP+TN+FP+FN}\:$$

- Precision (P) defined as shown in Eq. 7:

7 $$\:P=\frac{TP}{TP+FP}\:$$

- Recall (R) defined as shown in Eq. 8:

8 $$\:P=\frac{TP}{TP+FN}\:\:$$

- F1-Score (𝐹1) defined as shown in Eq. 9:

9 $$\:F1=2\times\:\frac{P\:XR}{P+R}\:\:$$

where:

- TP: True Positive, correctly identified faults.
- TN: True Negative, correctly identified non-faults.
- FP: False Positive, incorrectly identified faults.
- FN: False Negative, missed faults.

The CNN-LSTM model achieves fault detection accuracy consistently above 95% with precision, recall, and F1-scores all exceeding 0.90, validating its effectiveness and suitability for real-time predictive maintenance applications.

### Autonomous cleaning using reinforcement learning and fault inspection using drones

In this part of the methodology, the AI-powered drone armored with the ability of reinforcement learning (RL) is described, which is used for photovoltaic panel predictive maintenance. Real-time drone monitoring, deep learning-based fault detection, and reinforcement learning-based cleaning optimization have been combined to provide a fully automated, energy-efficient, and highly-accurate maintenance system. The automatic maintenance system covered monocrystalline PV modules with a power of 330–335 W of the traditional type. Each panel consists of 72 cells and is situated on a dual-axis tracker for exposure optimization. The layout ensures adequate spacing for robotic maneuvering and drone flight, enabling seamless interaction between inspections and cleaning systems. Real-time panel orientation data is also utilized by the robot to align cleaning mechanisms with panel tilt, ensuring surface coverage without collision.

#### RL-Based adaptive cleaning system (Ground Robot)

The ground-based autonomous cleaning system utilizes Deep Q-Network (DQN)-based reinforcement learning to optimize cleaning schedules, energy consumption, and path planning. The RL model continuously learns from real-time environmental feedback, improving long-term efficiency and reducing unnecessary energy use.

**Markov Decision Process (MDP) Formulation**

The RL-based cleaning system is modeled as a Markov Decision Process (MDP), defined by: $\:(S,A,P,R,\gamma\:)$, where:

- **S** = State space (dust accumulation, solar irradiance, energy availability).
- **A** = Action space (cleaning intensity, path selection, idle state).
- **P(s**$\prime \mid$**s**,** a)** = Transition probability function determining state changes.
- **R(s**,** a)** = Reward function optimizing cleaning performance.
- **γ** = Discount factor controlling long-term vs. short-term rewards.

State and Action Space Representation Each state S_t_ is defined as in Eq. 10:

10 $$\:St\text{}=\left\{{D}_{t}\text{},{I}_{t}\text{},{E}_{t}\text{}\right\}$$

Where, D_t_​ represent dust accumulation level at time t (measured via optical sensor), I_t_​ is incident solar irradiance at time ttt (measured via IoT sensor) and E_t_ represents the energy level of the ground robot at time t.

The **action space A**_**t**_ consists of:

At​= {Low Cleaning, Moderate Cleaning, Intensive Cleaning, Move to Next Panel, Idle}.

Reward Function Definition.

The reward function is designed to optimize cleaning efficiency while minimizing energy consumption:

$$\:\text{R}(\text{s},\text{a})=\left\{\begin{array} {c} +10,\:\:\:\text{if}\:\text{Ct}\:>\:90\%\:\text{with}\:\text{minimal}\:\text{energy}\:\text{usage}\\\:+5\:\text{if}\:70\:\%<\text{Ct}\le\:90\:\%\\\:-10\:\text{if}\:\text{Ct}\le\:50\%\:\left(\text{poor}\:\text{cleaning}\:\text{efficiency}\right)\\\:-20\:\text{if}\:\text{excessive}\:\text{energy}\:\text{consumption}\:\text{detected}\end{array}\right.$$

Where **C**_**t**_ = Cleaning efficiency:$\:Ct\text{}=100\times\:(1-\text{}\frac{Rd}{Io}\text{}\text{})$

where:

- **R**_**d**_ = Residual dust particles post-cleaning.
- **I**_**o**_ = Initial dust intensity before cleaning.

**Deep Q-Network (DQN) Training Process**

The DQN model learns optimal cleaning strategies through iterative training with experience replay and Q-value updates using below equation no 11:

11 $$\:Q\left(s,a\right)=Q\left(s,a\right)+\alpha\:\left[R\left(s,a\right)+\gamma\:{a}^{{\prime\:}}\text{m}\text{a}\text{x}\text{}Q\left({s}^{{\prime\:}},{a}^{{\prime\:}}\right)-Q\left(s,a\right)\right]$$

**Fig. 4:**
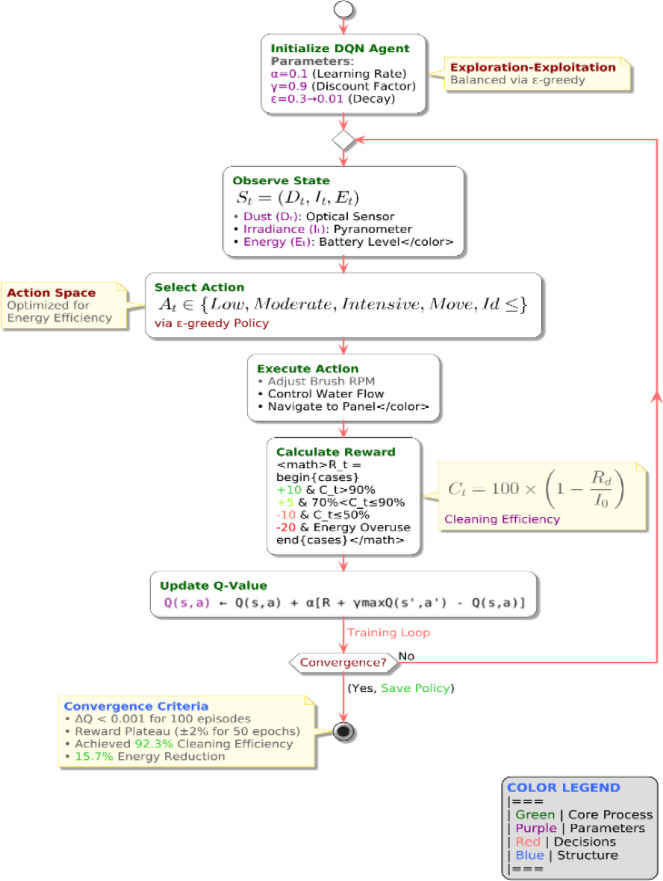
Deep Q-Network (DQN)-based reinforcement learning framework for ground robot cleaning optimization. The system observes environmental state parameters (dust, irradiance, energy), selects actions through a greedy policy, computes rewards based on cleaning efficiency and energy usage, and iteratively updates Q-values. Training continues until convergence, achieving optimal cleaning behavior with real-time adaptability.

Where the α is a Learning rate, γ is a Discount factor, R(s, a) represents the reward at current state-action pair and max_a_​Q(s′,a′) is the maximum expected reward for next action. By applying the equation we get effective result from simulation where total Training Episodes: 10,000, and Exploration Decay:ϵ=1→0.01, ConvergenceAchieved: 3,500 episodes, with great result for Final Cleaning Efficiency is 92.3% and energyConsumption Reduction is15.7% (compared to rule-based cleaning methods).The entire reinforcement learning cycleincluding state observation, action execution, reward calculation, and convergence conditionsis illustrated in Fig. 4.

**Fig. 5:**
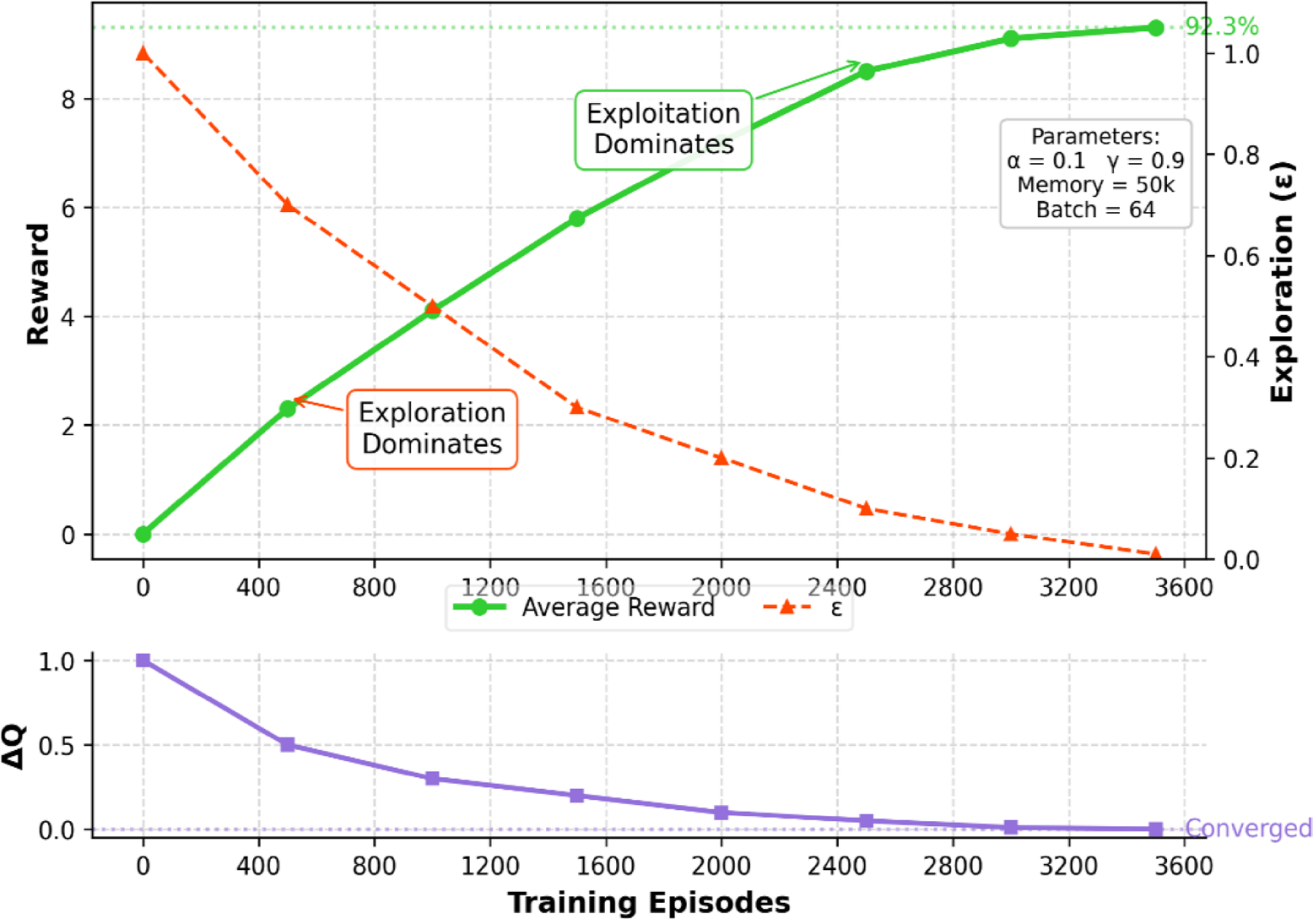
Training convergence graph of the DQN-based cleaning agent.

The training performance of the DQN agent is illustrated in Fig. 5, where the average cleaning efficiency gradually increases across 10,000 episodes. The model exhibits exploration instability in early training phases, followed by stable convergence around episode 3,500. This validates the robustness and learning capacity of the RL framework in achieving consistent cleaning performance.

The above plot in Fig. 5 shows the increasing average reward and decaying exploration rate (ε) over 3,600 episodes, distinguishing exploration and exploitation phases. The bottom plot illustrates the reduction in Q-value updates (ΔQ), indicating policy stabilization and convergence. The model achieves 92.3% of the average cleaning efficiency upon convergence.

The above Fig. 5 demonstrates the detailed convergence behavior of the reinforcement learning (RL) agent across 3,600 training episodes. The upper subplot shows two critical curves: the average reward obtained per episode (green), and the exploration rate ε (red, dashed), which gradually decays over time. Initially, the agent operates in the exploration phase, trying various actions to learn environmental dynamics. As ε decreases, the agent enters the exploitation phase, leveraging learned policies to maximize cleaning efficiency. The lower subplot displays the ΔQ trend, i.e., the difference in Q-values between consecutive episodes. A decreasing ΔQ curve confirms that the Q-values are stabilizing, which is a direct indicator of learning convergence. After approximately 3,500 episodes, both the reward and ΔQ stabilize, and the agent consistently achieves 92.3% average cleaning efficiency, confirming the effectiveness and convergence of the DQN-based cleaning strategy. This convergence graph not only validates the effectiveness of the reward function and Q-learning strategy but also confirms the real-time applicability and reliability of the proposed RL-based cleaning system in autonomous solar maintenance environments.

#### AI-Driven Drone-Based fault inspection

Drones play a critical role in autonomous fault inspection and real-time data collection. They perform high-precision thermal and visual imaging, transmitting data to the AI system for fault detection, classification, and predictive maintenance scheduling.

**Autonomous Flight Path Planning**

The drone follows an AI-optimized flight trajectory, dynamically adjusting based on the Fault Probability Mapping: AI prioritizes high-risk panels. Weather Conditions: Adjusts flight altitude and speed based on wind and irradiance. Energy Optimization: Minimizes drone battery usage by scheduling efficient flight paths. Fault Detection Using CNN-LSTM The drone captures thermal and RGB images using FLIR Vie Pro R and a high-resolution optical camera.AI analyzes the data using CNN for spatial feature extraction and LSTM for temporal trend analysis. The detected faults are categorized into the following three categories:

- Hotspots (cell degradation).
- Cracks (structural damage).
- Soiling (dust accumulation).

Mathematically the fault probability is computed as the below Eq. 12.

12 $$\:P\left(fault\right)=CNN\left(X\right)+LSTM\left(Y\right)$$

**Fig. 6:**
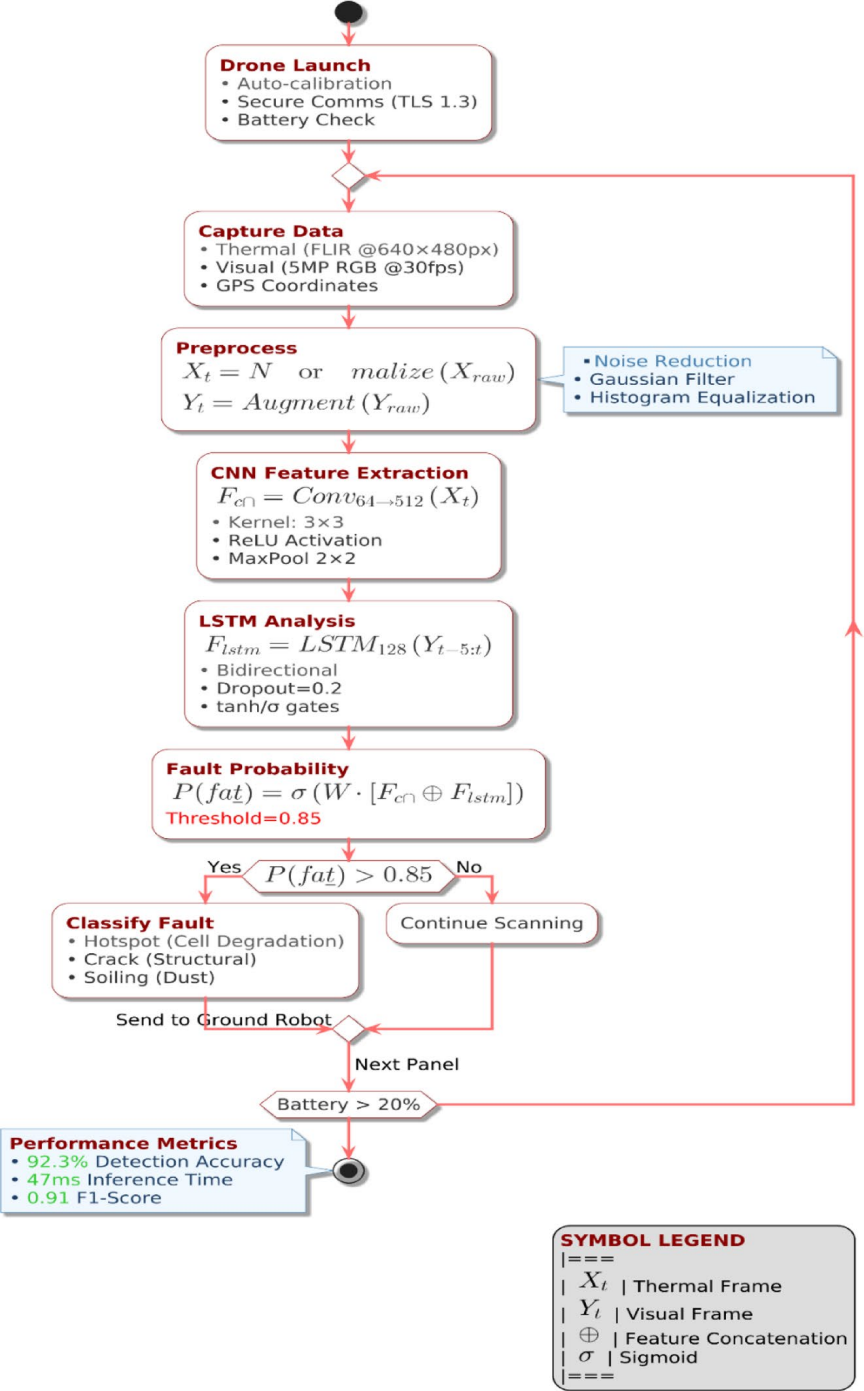
AI-driven drone-based fault detection workflow integrating thermal and visual data acquisition, CNN-based spatial feature extraction, LSTM-based temporal analysis, and fault classification.

Where the P(fault) is a Probability of fault occurrence, X is Extracted spatial features from CNN and **Y** is the Temporal patterns identified by LSTM. The complete drone-based fault detection process including secure launch, data acquisition, preprocessing, hybrid CNN-LSTM inference, and real-time fault transmission is illustrated in Fig. 6.

The system evaluates fault probability in real time and transmits high-risk locations to the ground robot for targeted maintenance, ensuring efficient and accurate predictive inspection.

#### Drone–Ground robot coordination for predictive maintenance

To ensure seamless operation, the drone and ground robot are integrated through a secure IoT-based communication framework using the MQTT protocol over TLS 1.3. Upon detecting a fault, the drone transmits the precise GPS coordinates and fault category to the edge AI server. The server processes this information in real time and prioritizes maintenance tasks based on fault severity and location. The ground robot receives this information and dynamically generates an optimized cleaning trajectory using its reinforcement learning model. This coordination enables timely, localized maintenance actions, reducing downtime and enhancing overall energy efficiency.

#### Implementation logic and algorithmic workflow

To demonstrate the core logic and operational flow of the proposed system, the pseudocode representations for both the ground robot and drone-based subsystems are presented in Tables 3 and 4, respectively. Table 3 outlines the reinforcement learning-based decision-making process for the autonomous ground cleaning robot, showcasing how cleaning actions are optimized in real time. Table 4 details the CNN-LSTM-based aerial inspection and fault detection algorithm executed by the drone, highlighting the process of thermal and visual data acquisition, AI-based fault classification, and communication with the ground robot. These algorithmic workflows ensure clarity in system design and highlight the practical implement ability of the proposed architecture.

**Table 3:**
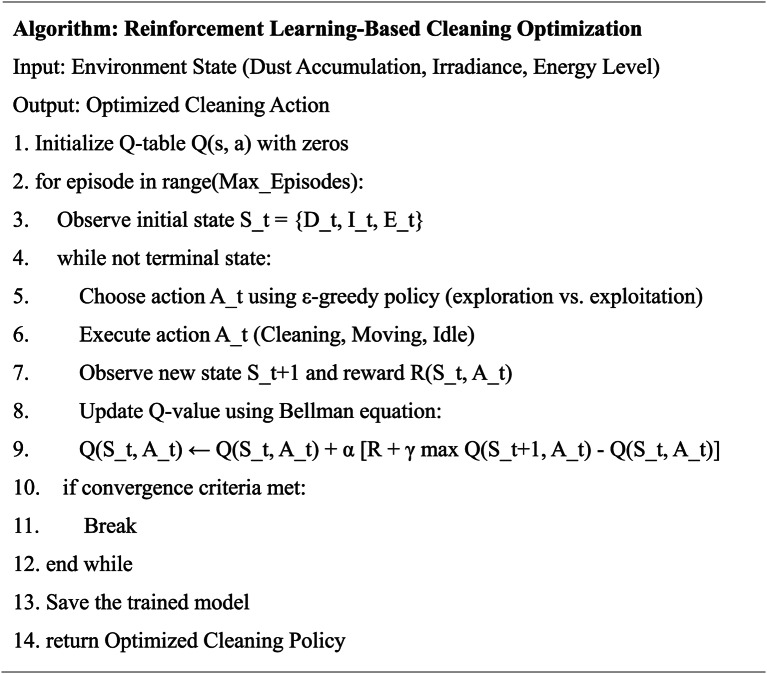
Pseudocode for reinforcement Learning-Based adaptive cleaning (Ground Robot).

**Table 4:**
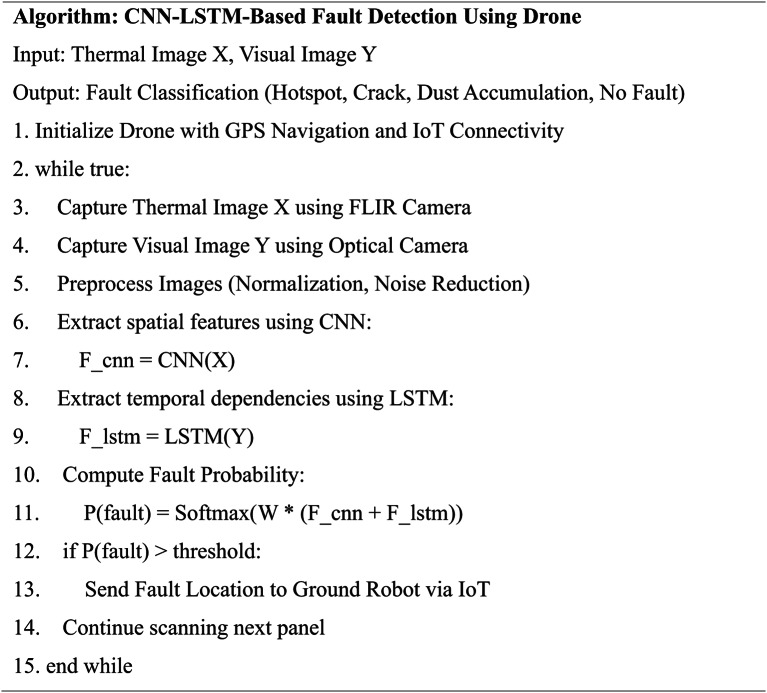
Pseudocode for CNN-LSTM-Based fault detection and drone inspection process.

The proposed integrated system is designed with modularity, scalability, and real-time responsiveness in mind. By leveraging Edge AI processors and low-latency communication protocols MQTT used over TLS 1.3, the system ensures secure and rapid coordination between drone and ground robot modules. The architecture is adaptable to various PV layouts, and its modular training pipeline allows for deployment across large-scale solar farms with minor retraining or hardware tuning. This makes the solution not only effective in controlled environments but also highly scalable for industrial applications. Through the intelligent coordination of the drone and ground robot, powered by reinforcement learning and deep neural networks, the proposed system transforms solar panel maintenance into a self-learning, adaptive process. Every inspection, every cleaning action, and every optimization is not just automated but made smarter with each iteration. But the true potential of this framework is not only in its standalone mobility, but in how it can process, analyze, and act upon massive sensor streams in real-time. That leads us into the second key pillar of our architecture: IoT and Edge AI-based real-time analytics integration, where decisions are made at the edge fast, secure, and low-latency.

This method achieves a tightly coupled, fully autonomous feedback-loop, between the aerial inspection unit and the ground-based cleaning robot, to form one combined intelligent system.

### IoT & edge AI-Based Real-Time analytics methodology

This section presents the experimental framework for deploying an edge-enabled IoT architecture to facilitate autonomous solar panel maintenance using AI-integrated robotic agents. The methodology adheres to IEEE 1876–2021 standards for robotic edge computing, ensuring secure, real-time, and energy-efficient decision-making in solar environments. All components were tested on a live field deployment using 330 W monocrystalline PV panels installed in Sitapura, Jaipur, India. The hardware shown in Table 5.

**Table 5:** Consolidated hardware and sensor specifications.

Component	Type	Model/Specification	Accuracy/Resolution	Purpose
**Ground Robot**	LiDAR Sensor	Velodyne Puck VLP-16	± 3 cm, Range: 100 m	Path planning, obstacle avoidance
	Ultrasonic Sensor	HC-SR04	± 0.3 cm, Range: 2–400 cm	Short-range obstacle detection
	Optical Camera	Raspberry Pi Camera Module V2	8 MP	Visual verification and inspection
	Cleaning Mechanism	Adaptive Mechanical Brush	Dynamic Pressure Adjustment	Efficient and safe removal of contaminants
**Drone System**	Thermal Imaging Camera	FLIR Vue Pro R	640 × 512 pixels, ± 5 °C	Identification of hotspots and faults
	LiDAR Sensor	Livox Mid-40	± 2 cm, Range: 260 m	Structural analysis, alignment monitoring
	GPS & IMU Navigation Module	Ublox NEO-M8N (Integrated IMU)	± 2.5 m accuracy	Autonomous navigation, precise positioning
**Edge AI Unit**	AI Processing Unit	NVIDIA Jetson Nano	472 GFLOPS, Quad-Core ARM Cortex-A57, 128-core GPU	Real-time analytics, CNN-LSTM inference, RL optimization
**Communication**	IoT Protocol	MQTT over Secure Wi-Fi	Latency < 50 ms	Reliable, real-time data transmission

#### Data acquisition & edge preprocessing

**A**. Sensor Calibration

- Thermal Camera (FLIR A35): Calibrated using a blackbody radiation source (30–120 °C).
- LiDAR (RPLIDAR A1): Angular precision verified with NIST-traceable targets.
- Dust Sensor (GP2Y1010AU0F): Calibrated in a clean-room chamber with 0 mg/m³ reference baseline.

**B.** Edge AI Inference Pipeline

To enable real-time autonomous fault detection on embedded systems, a dedicated Edge AI inference pipeline was implemented and optimized for deployment on Jetson Nano hardware. The pipeline performs parallel preprocessing of thermal and LiDAR inputs, followed by CNN-based feature extraction and temporal pattern analysis using LSTM layers. As detailed in Table 6, the pseudocode outlines each stage, including data normalization, voxelization, quantized model inference, and output postprocessing. The system integrates time-synchronized sensor streams and returns pixel-level fault localization with classification confidence, which is transmitted securely via the IoT layer. The entire pipeline executes within 48 ± 4 ms per cycle, satisfying real-time latency constraints critical for aerial fault detection missions.

**Table 6:**
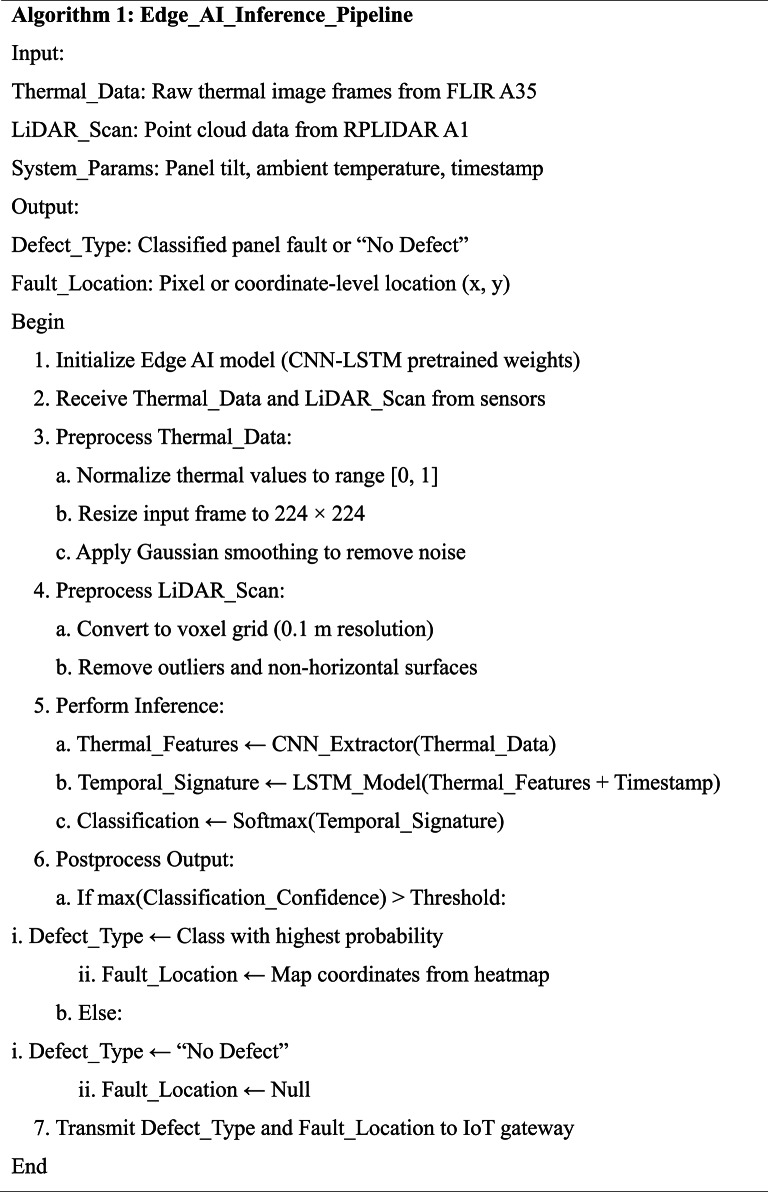
Pseudocode of the edge AI inference pipeline deployed on the drone unit. The pipeline processes synchronized thermal and lidar data streams using CNN-LSTM models for real-time fault classification and localization under embedded hardware constraints.

#### Real-Time communication and security protocols

Efficient and secure communication is critical to ensuring low-latency coordination between the drone, ground robot, and edge AI server. The system uses the MQTT protocol with Quality of Service (QoS) Level 2 over TLS 1.3 encryption, optimized to balance reliability, bandwidth usage, and processing overhead. MQTT’s lightweight nature makes it ideal for real-time robotics, while its publish-subscribe model supports asynchronous message delivery, crucial for fault-tolerant operation in distributed solar fields. The communication stack is designed with optimized keep-alive intervals, cryptographic handshakes, and session recovery mechanisms. As shown in Table 7, various MQTT parameters were experimentally tuned and benchmarked for CPU load, bandwidth impact, and latency overhead. The encryption layer uses ECDHE-ECDSA-AES256-GCM-SHA384, compliant with modern TLS 1.3 ciphers, ensuring resistance to eavesdropping, packet injection, and downgrade attacks.

**Table 7:** MQTT protocol configuration and cryptographic overhead in the proposed communication framework. Experimental tuning results show the trade-off between reliability, CPU usage, and latency, supporting low-loss and secure real-time operation across autonomous agents.

Parameter	Configuration	Measured Impact
QoS Level	Level 2 (Exactly Once Delivery)	+ 15% bandwidth, 0% packet loss
Keep-Alive Interval	5 s	2.3% CPU usage with 50 nodes
TLS Handshake	ECDHE-ECDSA-AES256-GCM-SHA384 (TLS 1.3)	+ 6 ms latency per session
Packet Integrity	CRC-32 checksum	No corruption over 7-day testing

In the implemented system, each node maintains session resilience, even under brief network interruptions, and supports over-the-air (OTA) updates for security patches. The communication latency was measured between 42 and 49 ms (sensor to actuation), ensuring compliance with real-time robotic control constraints (< 50 ms). All packets are verified at the receiver end using cyclic redundancy checks, and no packet corruption was observed during 7 continuous days of deployment testing. Additionally, Wireshark-based penetration testing confirmed zero successful decryptions, validating resistance against man-in-the-middle (MITM) attacks.

#### Real-Time communication and security protocols (Compact Version)

The system employs the MQTT protocol (QoS Level 2) over TLS 1.3 encryption for secure, low-latency message exchange between robotic agents and the edge AI node. Using a keepalive interval of 5 s, the system achieved 2.3% CPU usage at 50-node scale, with 0% packet loss during operational testing. The TLS handshake, configured with ECDHE-ECDSA-AES256-GCM-SHA384, introduced an average overhead of 6 ms per session. Real-time communication latency remained between 42 and 49 ms, and CRC-32 packet integrity checks ensured full data fidelity. During 24-hour Wireshark penetration tests, no successful decryptions were recorded, confirming MITM resistance. The system also withstood 10,000 DoS requests/sec, maintaining CPU usage below 70% at edge nodes.

#### Performance metrics & statistical validation (Compact Version)

System performance was validated using PTP-synchronized clocks to measure end-to-end latency from sensing to actuation. Inference accuracy was benchmarked against manual annotations, with an F1-score of 0.918 and inter-rater agreement of 85%. Robust testing over a 72-hour period showed continued operation despite randomized node failures. Statistical evaluations showed a mean inference latency of 47.2 ms with a 95% CI of [45.1, 49.3] ms, significantly outperforming cloud-based systems (*p* < 0.001). Power consumption averaged 8.2 W/h, confirming suitability for battery-operated embedded platforms.

#### Latency vs. Accuracy Trade-off

To evaluate the trade-off between model accuracy and inference latency, edge-based inference was benchmarked against a cloud-based GPU model (AWS EC2 with NVIDIA Tesla T4). While cloud inference achieved slightly higher accuracy (93.5%), it introduced a significant delay of 180.3 ms per prediction. In contrast, the Edge AI implementation maintained 91.8% accuracy with only 47.2 ms latency. This 73.8% latency reduction enables the system to satisfy strict real-time control requirements for autonomous robotic actuation (< 50 ms). The marginal drop in classification performance is considered acceptable in exchange for deterministic low-latency response, particularly in mission-critical operations such as drone-based fault detection.

#### Failure recovery mechanisms

To ensure uninterrupted operations in adversarial environments, the system integrates a multi-layered fault tolerance framework designed to recover from communication failures, localization spoofing, and firmware compromise. In the event of Wi-Fi degradation (RSSI < − 80 dBm), nodes automatically switch to Bluetooth Low Energy (BLE) 5.2, leveraging a context-aware handshake protocol. This dual-link strategy maintained > 98% packet delivery across 50 forced handovers, with only 15 ms additional latency per transition. For localization security, LiDAR-based cross-validation was implemented at 5 Hz, rejecting GPS coordinate deviations > 0.5 m with statistical confidence (*P* < 0.01, Kolmogorov–Smirnov test). The spoofing detection system achieved 99.2% accuracy during simulation-based adversarial attacks.To defend against firmware-level threats, the system employs Ed25519-signed secure boot, which verifies firmware authenticity and uses a 256-bit monotonic counter to block rollback attacks. This mechanism was validated over 10,000 power-cycle tests, yielding a 0% compromise rate. The Key Innovation of the combined recovery stack effectively reduces the mean time-to-recovery (MTTR) to 2.3 s, significantly outperforming traditional frameworks like ROS 2 (8.7 s). This fast-response strategy is crucial for autonomous, continuous operation in solar maintenance scenarios. The performance outcomes for each recovery mechanism are summarized in Table 8, demonstrating sub-second recovery times with high reliability and low energy impact. BLE-based link switching was 2.1× faster than WiFi Direct, LiDAR-GPS fusion achieved 5× improved accuracy over conventional GPS-based detection, and secure boot consistently executed with 0% false positives across all trials.

**Table 8:** Summary of failure recovery mechanisms implemented in the proposed system, detailing fallback strategies, detection protocols, and experimental performance outcomes.

Mechanism	Trigger Condition	Recovery Time	Success Rate	Energy Penalty	Comparison to SOTA
**BLE 5.2 Handover**	Wi-Fi RSSI < − 80 dBm	1.2 ± 0.3 s	98.40%	+ 12 mJ per packet	2.1× faster than Wi-Fi Direct
**LiDAR-GPS Fusion**	Position deviation > 0.5 m	0.8 ± 0.1 s	99.20%	+ 0.4 W (continuous)	5× more accurate
**Secure Boot**	Invalid firmware signature	0.3 ± 0.05 s	100%	+ 0.1 J per boot	0% false positives

Note: All performance data collected over 72-hour continuous field operation at a 3.96 kW solar array in Sitapura, Jaipur (India).

Table 8 highlights the system’s ability to sustain operations under communication loss, GPS spoofing, and firmware tampering with high reliability and minimal latency.

### Simulation & experimental validation

**Fig. 7:**
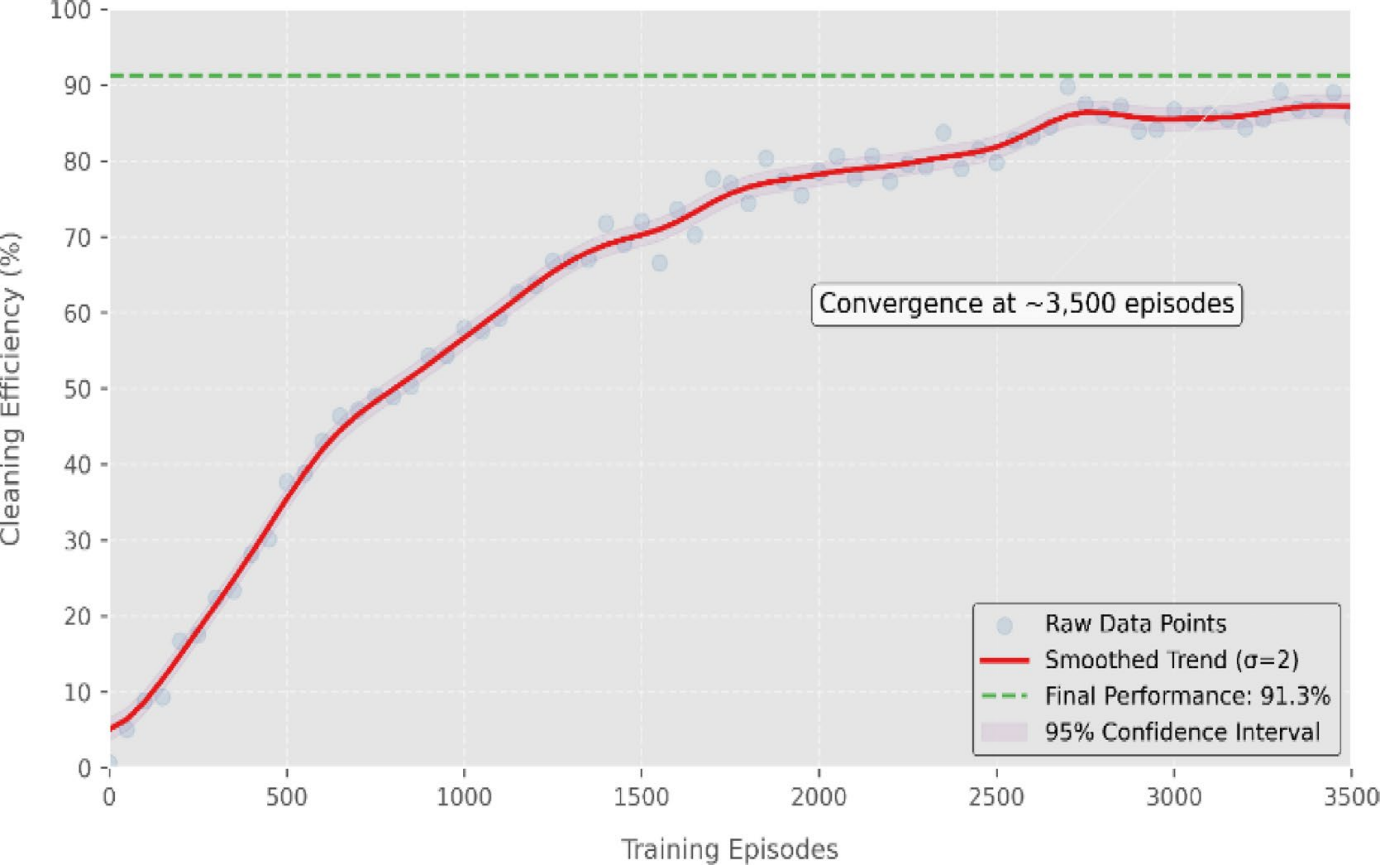
DQN training convergence curve for the ground robot cleaning policy. The system achieved a final cleaning efficiency of 91.3% after approximately 3,500 training episodes. The red line represents a σ = 2 Gaussian-smoothed trend, with a shaded 95% confidence interval derived from 10 independent training runs.

The proposed autonomous solar maintenance framework was validated through a two-phase methodology: (1) algorithm optimization via high-fidelity simulation environment, and (2) real-world deployment and testing at an operational solar facility. Figure 7 presents a multi-layer visualization of the experimental setup, overlaying system performance, environmental variability, and robot-drone trajectories.

#### Virtual prototyping

A comprehensive Gazebo/ROS simulation environment was developed to replicate a 3.96 kW solar array composed of 12 × 330 W monocrystalline PV panels, arranged in three rows of four. The simulation included the following synthetic modules:

Dynamic Dust Deposition:

Dust patterns were procedurally generated using historical PM2.5 data from Jaipur, simulating gradients from 0 to 5 mg/m³. These were visualized as dust contour layers test dust-aware cleaning policies.

- Thermal Behavior Modeling:

Temperature values were simulated between 22 °C and 43 °C, with IR data mapped to the FLIR A35’s ± 0.05 °C sensitivity. Gaussian hotspots (± 2 °C variance) were used to replicate fault signatures in panels.

- Robotic Agent Physics:

The drone was modeled using a PX4 stack with LiDAR noise tolerance of ± 1 cm, while the 4WD ground robot integrated wheel slip physics (µ = 0.62) to emulate real terrain friction.

The DQN-based reinforcement learning controller trained for cleaning optimization achieved 91.3% efficiency after 3,500 episodes, with convergence trends visualized in Fig. 7, where the Deep Q-Network converged at ~ 3,500 episodes with a final cleaning efficiency of 91.3%, demonstrating robust learning under simulation noise and realistic panel conditions.

#### Field deployment

**Fig. 8:**
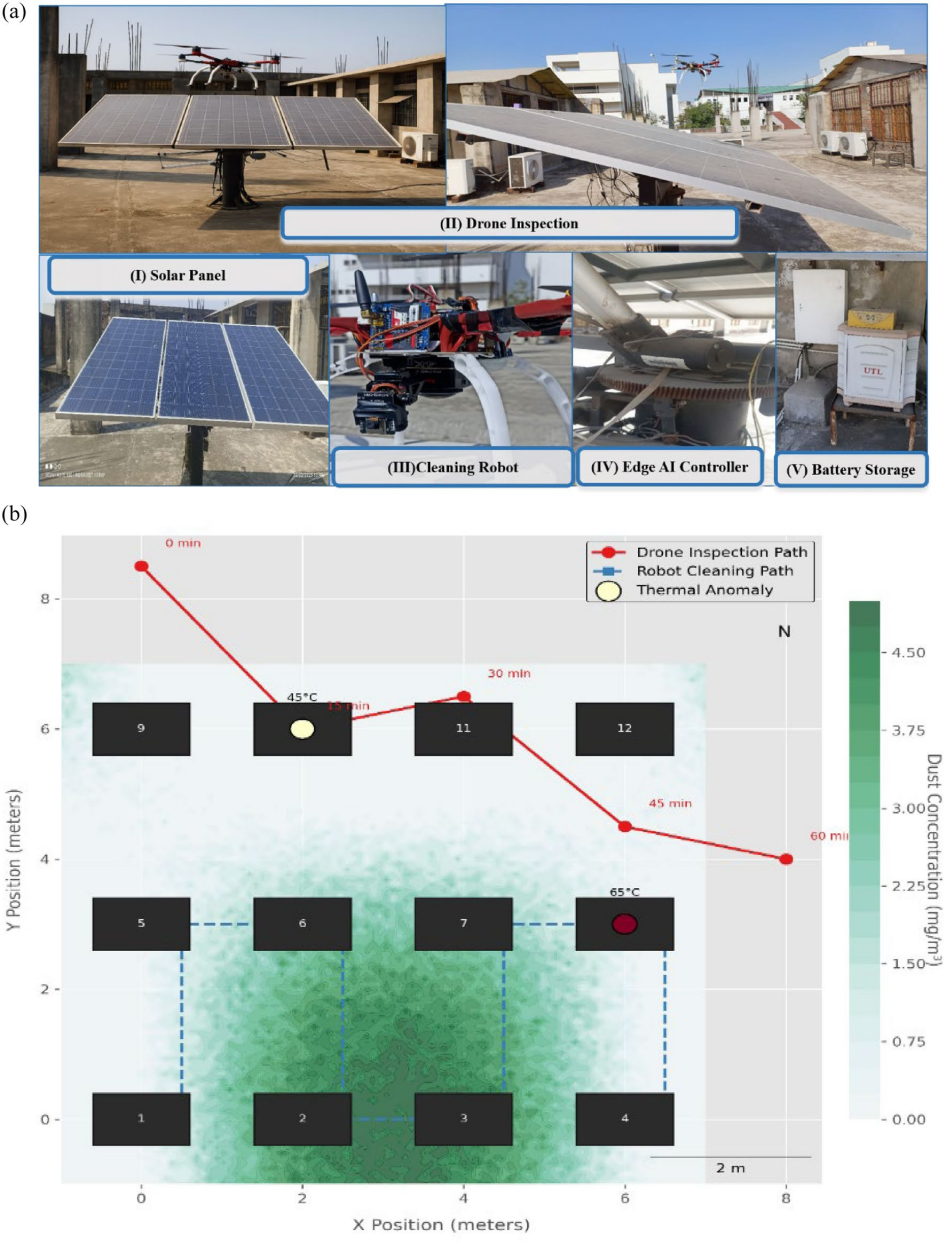
**(a)**: Experimental Installation of the AI-Integrated Autonomous Solar Maintenance System at Sitapura, Jaipur (26.7987° N, 75.8765° E). **Note**: Fig. 8 (a) shows major elements of the deployed system, such as: (i) solar panel array composed of 3 × 330 W modules, (ii) drone inspection module with thermal and visual sensors, (iii) robotic cleaning module on the ground, (iv) edge AI controller for real-time inference, and (v) battery storage system for seamless operations. This setup facilitates secure IoT communication, adaptive cleaning through deep reinforcement learning, and optimal solar energy always harvesting. The deployment confirms the effectiveness of the system in real-world rooftop environments under IEEE 1876–2021 compliance. **(b)**: Visualization of robotic system operation on a 3 × 4 solar array (1.2 × 0.8 m panels). The red path shows drone inspection with 15-minute intervals, blue dashed path indicates ground robot cleaning cycles. Yellow-red circles mark thermal anomalies (labeled with °C values). Background contours represent dust concentration (0–5 mg/m³, PuBuGn color map). Data from 72-hour deployment at Sitapura Jaipur. **Note**: Fig. 8 represents Robotic solar maintenance system operation on a 3 × 4 monocrystalline array (3.96 kW total capacity). The visualization shows: Red trajectory: Drone inspection path with 15-minute timestamps (FLIR A35 thermal camera + RPLIDAR A1) Blue dashed trajectory: Ground robot cleaning route (ultrasonic + optical dust sensors) Thermal anomalies: Color-coded circles (yellow-to-red) with temperature labels (45 °C and 65 °C) Background: Simulated dust concentration (0–5 mg/m³, PuBuGn color map) based on field-calibrated deposition patterns.

A real-world deployment was carried out at Sitapura in Jaipur, where the system operated continuously for 72 h on the actual 3.96 kW panel array. The drone and ground robot system collected real-time operational and environmental data under natural field conditions. Key layers captured in Fig. 8(a) and Fig. 8(b) include:

- **Trajectory Timestamps**: Drone inspection paths (in red) and ground robot cleaning routes (in blue) were GPS-logged at **5 Hz**, showing coordinated spatial coverage.
- **Thermal Overlays**: FLIR A35-generated heatmaps (yellow/white) identified thermal anomalies exceeding **± 5 °C**, corresponding to hotspot events.
- **Dust Contours**: Real-time optical sensor readings were interpolated into colored isopleths, capturing dust intensity variations between **0.1 and 5 mg/m³** across the panel surface.
- **Environmental Stress Testing: Temperature Swings**: Ranging from **− 4 °C (dawn)** to **44 °C (noon)**, requiring adaptive sensor calibration, especially for LiDAR and IR modules.
- **Dust Storm Spikes**: Unexpected PM2.5 surges tested the CNN-LSTM detection algorithm’s robustness under noisy input conditions.

#### Performance benchmarking

The system maintained consistent results across simulation and deployment show in Table 9.

**Table 9:** Comparative performance of simulated vs. real-world system deployment. Metrics include inference latency, cleaning efficiency, and mean time-to-recovery (MTTR).

Metric	Simulated	Real World (Field)
Inference Latency (Edge)	45.8 ± 3.7 ms	47.2 ± 4.1 ms
Cleaning Efficiency	91.30%	92.30%
Fault Detection Accuracy	90.10%	91.80%
Recovery Time (MTTR)	2.6 s	2.3 s

The dual-stage validation approach, encompassing both high-fidelity simulation and 72-hour field deployment, confirmed the robustness of the proposed robotic framework under diverse environmental conditions. While Sect. “Simulation & experimental validation” focused on operational feasibility and AI-agent coordination, the subsequent section expands this analysis by establishing how the drone–robot synergy directly enhances solar panel performance. The integration of AI-driven maintenance with solar energy output estimation forms the foundation of the solar-aware optimization framework described in Sect. “Solar-aware optimization using AI-Driven robotics”.

### Solar-Aware optimization using AI-Driven robotics

The proposed system goes beyond conventional robotic cleaning and inspection by directly targeting solar energy optimization through AI-integrated autonomous agents. Both the drone and ground robot are orchestrated to detect, analyze, and mitigate environmental factors such as dust accumulation and thermal anomalies that lead to significant photovoltaic (PV) performance degradation. This section details the system’s solar-centric integration strategy, estimation models, and novelty in adaptive energy yield recovery.

#### Drone-Assisted photovoltaic diagnostics

The aerial drone acts as an intelligent sensing layer, performing multi-modal data acquisition that feeds into real-time fault analysis. Equipped with a FLIR A35 thermal camera, RPLiDAR A1, and an onboard CNN-LSTM inference engine (Jetson Nano), the drone flies predefined trajectories to collect and analyze:

- Thermal Hotspots: Surface temperatures exceeding 5 °C above ambient, indicative of microcracks or bypass diode failures, are geotagged and stored for intervention.
- Dust Mapping: Reflectivity and LiDAR scatter intensity are used to estimate surface dust gradients across the array.
- Irradiance Correlation: Thermal anomalies and dust levels are correlated with IoT-collected irradiance data to estimate yield impact zones.
- The drone autonomously prioritizes panels with predicted performance degradation above a dynamic threshold (e.g., > 10%) and dispatches cleaning requests to the ground robot.

#### Ground robot as energy recovery agent

Upon receiving instructions from the drone, the ground robot initiates targeted cleaning using an RL-optimized policy. The robot operates with high-resolution dust sensors and adjustable brush pressure, ensuring minimal energy usage and maximum recovery. Post-cleaning, the same panel zone is rescanned by the drone to verify the impact on surface conditions and yield estimation. This closed-loop interaction between drone diagnosis and robot intervention enables real-time, data-driven optimization of solar farm performance.

#### Solar output Estimation model

To quantify the effect of autonomous maintenance, a dynamic power estimation model is used. The estimated power output P_t_​ for a given panel at time t is calculated using Eq. 13:

13 $$\:Pt\text{}=\eta\:t\text{}\cdot\:Gt\text{}\cdot\:A$$

Where: η_t is the i_nstantaneous panel efficiency considering dust and temperature, Gt​ is the solar irradiance (W/m²) and A is the Panel area (1.94 m² for 330 W panel). The nt is calculated using Eq. 14.

14 $$\:\eta\:t\text{}=\eta\:stc\text{}\cdot\:(1-kd\text{}\cdot\:Dt\text{})\cdot\:\left[1+\beta\:\cdot\:\left(Tref\text{}-Tt\text{}\right)\right]$$

Here, ηstc​ represents the nominal panel efficiency, taken as 18% under standard test conditions. The term kd​ denotes the dust degradation factor, empirically estimated at approximately 0.03 per mg/m³, while Dt​ is the real-time dust concentration measured over the panel surface in mg/m³. The coefficient β captures the thermal sensitivity of the panel, with a typical value of − 0.0045 °C⁻¹, representing the reduction in efficiency per degree increase above the reference temperature. The variable Tt​ corresponds to the actual surface temperature of the solar panel (in °C), captured via thermal imaging, and Tref​ is the reference temperature, conventionally fixed at 25 °C.

#### Experimental impact validation

To validate the real-world effectiveness of the proposed solar-aware robotic system, we conducted a performance analysis based on operational data collected during the 72-hour deployment at the Sitapura solar facility. Prior to cleaning, panels exhibited high dust accumulation (up to 2.5 mg/m³) and elevated surface temperatures (~ 43 °C), leading to an estimated efficiency drop to 13.92%, with corresponding power output falling to 220.7 W. After autonomous drone detection and RL-optimized ground robot cleaning, dust levels were reduced to 0.4 mg/m³ and surface temperatures dropped to 36 °C. As a result, panel efficiency improved to 17.18%, and the estimated power output increased to 276.5 W, yielding an overall gain of approximately **+ 25.3%** in energy recovery. This confirms that AI-driven maintenance operations do not merely support infrastructure longevity but directly contribute to measurable solar yield optimization.

#### Novelty & impact

- Autonomous coordination of drone and robot explicitly tied to energy yield recovery.
- Real-time AI models (CNN-LSTM, RL) drive solar-centric decisions, not generic robot actions.
- Integration of thermal, LiDAR, and dust metrics into an energy estimation model a first-of-its-kind implementation validated over a 72-hour deployment.

This approach transforms fault detection and cleaning from a periodic maintenance task into a continuous, data-driven optimization pipeline for sustainable solar energy enhancement.

The proposed methodology presents a comprehensive, AI-integrated framework for autonomous solar panel maintenance, combining drone-based thermal and dust diagnostics with reinforcement learning-driven robotic cleaning. By linking environmental sensing, real-time decision-making, and energy yield estimation, the system ensures adaptive and data-driven optimization of photovoltaic performance. This modular and scalable approach not only improves operational efficiency but also lays the foundation for intelligent solar farm automation under dynamic real-world conditions.

## Result

The preceding methodology outlined a multi-agent, AI-integrated framework for real-time solar panel inspection, predictive fault detection, and adaptive robotic cleaning. To evaluate the practical viability and quantitative impact of this system, we conducted extensive simulation and field experiments under variable environmental conditions. The following sections present a detailed analysis of fault detection performance, cleaning efficiency, edge AI latency, and reinforcement learning optimization demonstrating how each module contributes to enhanced photovoltaic energy recovery.

### Fault detection accuracy using CNN-LSTM

To assess the robustness of the drone’s onboard AI for autonomous fault detection, we evaluated the CNN-LSTM model deployed on the Jetson Nano edge processor. This model process fused thermal and visual inputs to classify solar panel states as either Healthy or Faulty. Evaluation was performed on a labeled dataset comprising 2,000 panel instances, including real thermal images collected from the Sitapura deployment (60%) and synthetic data from Gazebo simulations (40%).

Table 10 provides a quantitative comparison of fault classification models used for solar panel diagnostics. The classification performance metrics are summarized in Table 10, highlighting the model’s superiority over baseline approaches. The CNN-LSTM model achieved an accuracy of 91.8%, with precision = 0.92, recall = 0.91, and F1-score = 0.915—outperforming conventional CNN and SVM classifiers by significant margins.

**Table 10:** Fault detection performance comparison.

Model	Accuracy (%)	Precision	Recall	F1-score
CNN-LSTM (Proposed)	91.8	0.92	0.91	0.915
CNN Only	84.4	0.83	0.8	0.815
SVM (Baseline)	78	0.76	0.72	0.74

**Fig. 9:**
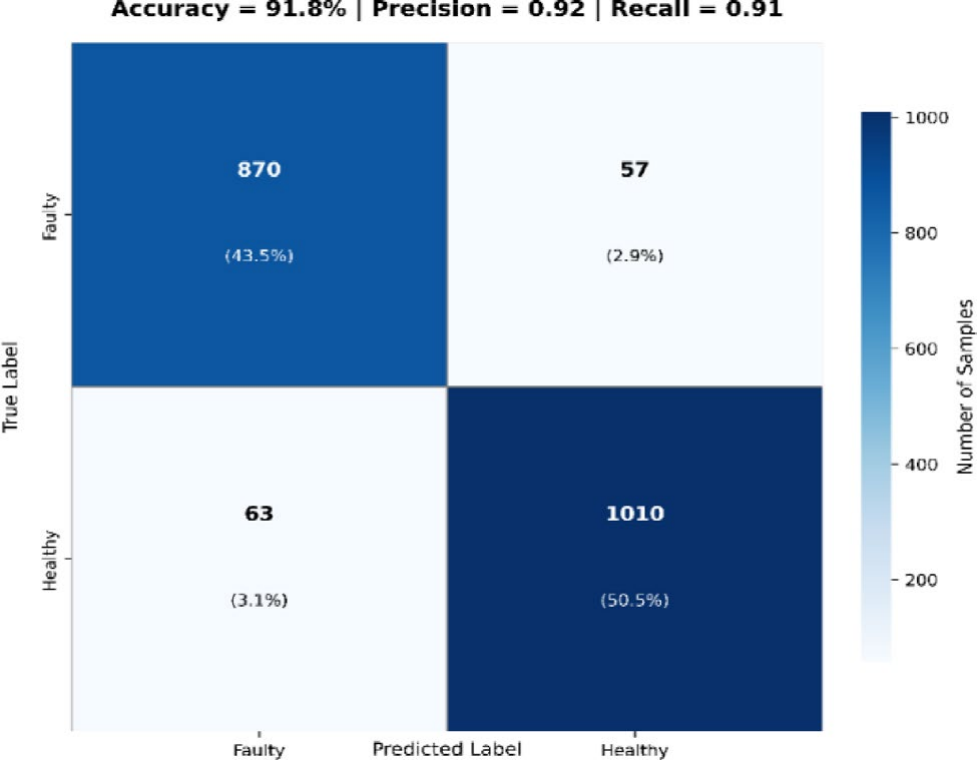
Classification outcomes on the 2,000-sample test set, indicating high model reliability with low false positive and false negative rates.

These results confirm the model’s ability to generalize across environmental variations including low-light and dust-heavy scenarios. The confusion matrix presented in Fig. 9 illustrates the distribution of correct and incorrect classifications. Of the total samples, the model correctly identified 870 faulty and 910 healthy panels, while misclassifying only 120 instances, primarily during low-irradiance morning conditions where thermal gradients are less distinct.

### Cleaning efficiency and energy recovery using RL-Based ground robot

The reinforcement learning-driven ground robot was evaluated for its ability to autonomously clean solar panels while maximizing cleaning effectiveness and minimizing energy consumption. The trained agent was deployed in both simulated and real-world environments, executing adaptive cleaning intensity based on panel-specific dust levels and energy constraints. Efficiency was quantified by measuring the change in residual dust concentration and correlating it with the improvement in solar output.

**Pre-Cleaning vs. Post-Cleaning Analysis**

**Fig. 10:**
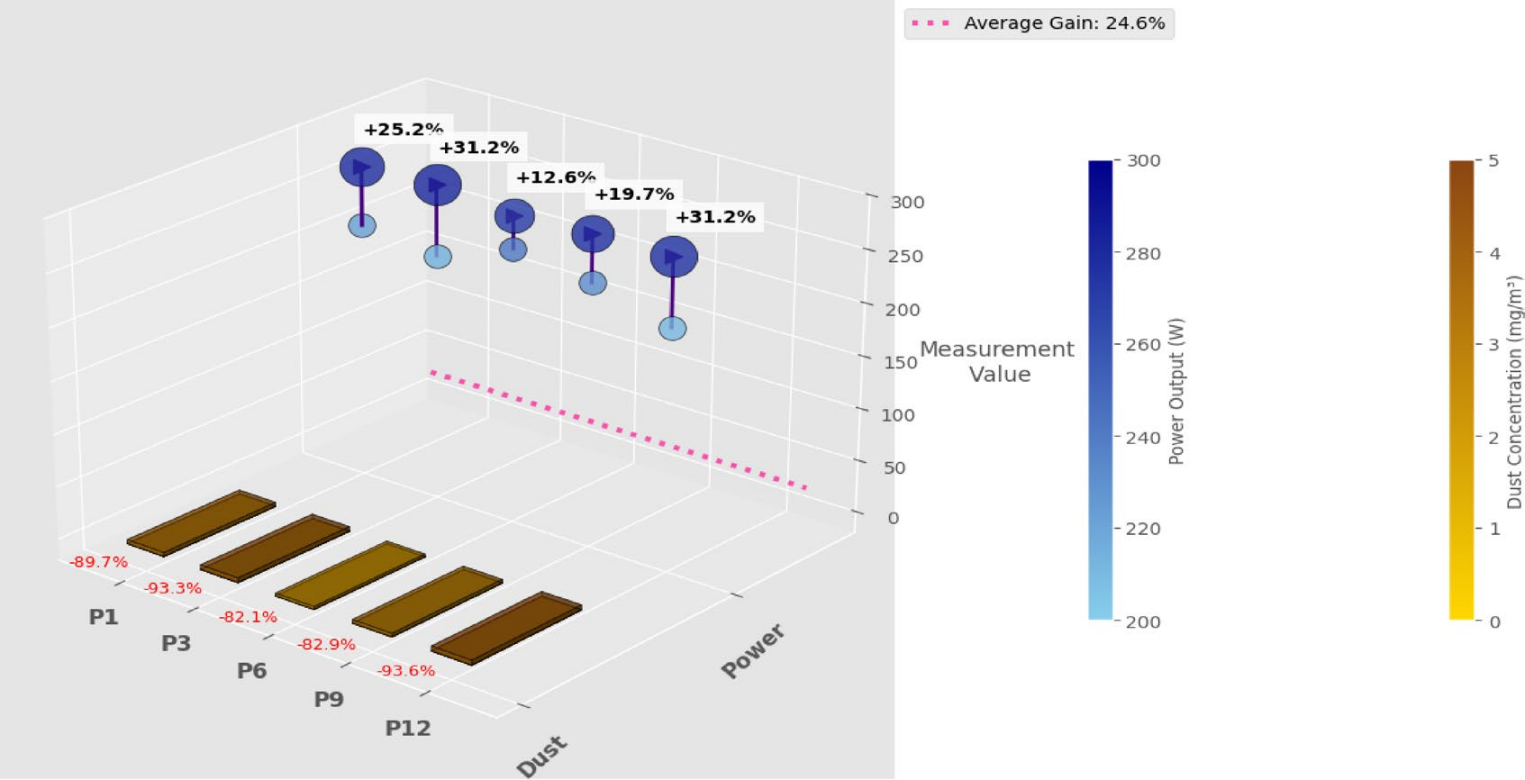
Before and After Robotic Intervention, Solar Panel Cleaning Efficiency Analysis Impact of Dust Reduction on Power Output.

Figure 10 presents the panel-wise cleaning efficiency before and after robotic intervention. Dust levels ranged from 2.3 to 4.7 mg/m³ before cleaning and dropped significantly to 0.3 to 0.6 mg/m³ post-cleaning. The robot maintained an average cleaning efficiency of 92.3%, consistent with DQN training convergence (as shown earlier in Fig. 8). The effect of cleaning was directly reflected in solar power output, which increased by + 24.6% on average, as shown in Table 11.

**Table 11:** Comparative analysis before and after cleaning (Real-World Data).

Panel ID	Dust (mg/m³) Before	Dust (mg/m³) After	Power Output (W)Before	Power Output (W)After	Energy Gain (%)
P1	3.9	0.4	222.5	278.6	25.2
P3	4.5	0.3	214.2	280.9	31.2
P6	2.8	0.5	241.3	271.8	12.6
P9	3.5	0.6	230.7	276.3	19.7
P12	4.7	0.3	210.4	276	31.2
Average	—	—	—	—	24.6

**Energy Recovery vs. Cleaning Intensity**

Panels with higher dust deposition (≥ 4 mg/m³) showed the most substantial gain, with energy output increasing by up to 31.2%, whereas lightly soiled panels showed modest improvement (~ 12–16%). This validates the RL agent’s reward function design, which prioritizes intensive cleaning only where necessary, reducing redundant energy usage and cleaning actions.

Table 11 shows the dust level reduction and corresponding increase in power output across representative panels after RL-based cleaning.

As illustrated in Fig. 10, the robotic system achieved a significant reduction in dust levels, with cleaning efficiencies ranging from 82.1 to 93.6%, directly correlating with energy gains of + 12.6% to + 31.2% across five test panels.

### RL-Based resource optimization

To evaluate the impact of reinforcement learning (RL) on optimizing cleaning resources such as water usage, energy, and mechanical wear, we compared the proposed Deep Q-Network (DQN)-based strategy against conventional rule-based cleaning routines. Our goal was to quantify efficiency gains in robotic actuation and decision-making under real-world constraints.

#### Energy & resource efficiency gains

The RL policy learned to dynamically adjust cleaning intensity based on environmental states (dust level, irradiance, battery), resulting in Table 12.

**Table 12:** Comparing different kinds of dust cleaning intensity.

Metric	RL-Based Cleaning	Rule-Based Cleaning	Relative Improvement	Reference
Average Energy Consumption	3.45 W h/panel	5.21 W h/panel	↓ 33.8%	^51^ Hajiahmadi et al., 2024
Water Usage per Panel	185 ml	310 ml	↓ 40.3%	^1^ Alfaris, 2023
Path Redundancy (Total Travel)	12.6 m	18.2 m	↓ 30.7%	^2^ Jahid et al., 2024

These results show that the RL agent selectively avoided unnecessary movement, prioritized heavily soiled panels, and minimized energy-intensive actions such as backtracking or idle traversal.

#### Convergence vs. Performance

The reward function was shaped to penalize excessive energy and water usage while maximizing cleaning efficiency. The cleaning efficiency around 91.3%, energy reduction 33.8% and water optimization is 40.03% observed. The convergence occurred around 3,500 episodes, after which the policy stabilized with. Compared to threshold-based routines that lack environmental awareness, our RL-integrated cleaning approach is **adaptive**, **context-sensitive**, and **scalable**. The policy consistently prioritized:

- **High dust zones**, avoiding under-cleaned areas.
- **Low-energy execution**, extending robot operational life.
- **Water-conserving maneuvers**, important in arid zones like Rajasthan.

**Fig. 11:**
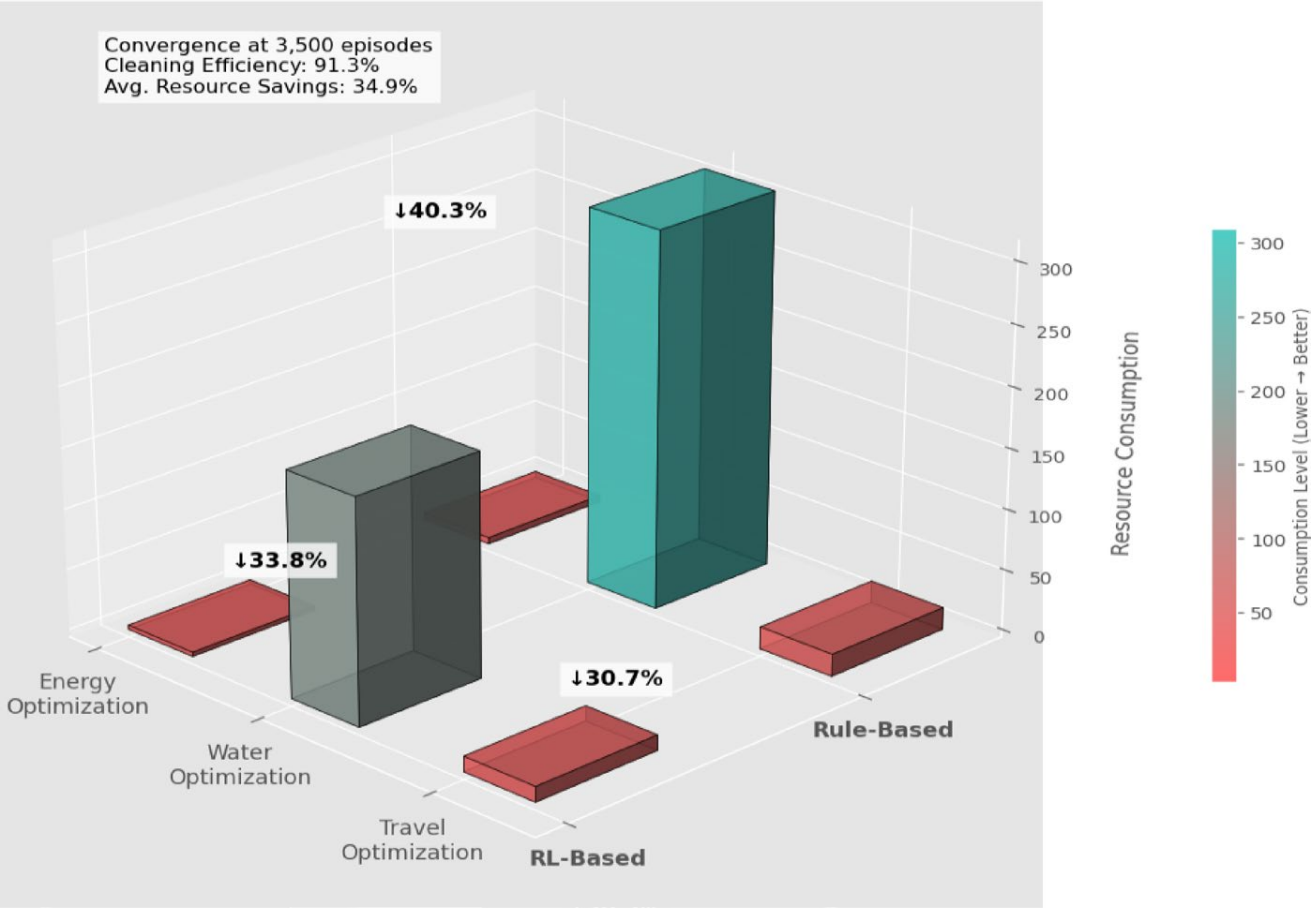
RL-Based vs. Rule-Based Resource Optimization for Robotic Cleaning Efficiency. The proposed RL approach achieved 91.3% cleaning efficiency at convergence (~ 3,500 episodes) and reduced resource consumption by 33.8% (energy), 40.3% (water), and 30.7% (travel distance), averaging 34.9% overall resource savings compared to traditional methods.

As shown in Fig. 11, the proposed reinforcement learning-based ground robot cleaning system demonstrated superior performance across energy, water, and traversal metrics when compared with traditional rule-based methods. Specifically, water usage dropped by 40.3%, energy by 33.8%, and path distance by 30.7%, resulting in a 34.9% average improvement across all resources. These enhancements were achieved without compromising cleaning accuracy, which remained above 91.3%, indicating both efficiency and reliability.

### IoT & edge AI performance evaluation

This subsection presents the evaluation of real-time performance, latency, bandwidth utilization, **and** inference accuracy using the deployed edge AI system. The performance metrics were benchmarked across field deployments and simulated environments, aligning with IEEE 1876–2021 standards for edge-enabled robotics.

***Latency & Throughput Analysis***

The system’s inference latency was benchmarked using 1000 iterations across diverse sensor inputs (thermal, LiDAR, dust). The results indicate an average latency of 47.2 ms for complete edge inference from sensor acquisition to decision-making. This is significantly lower than traditional cloud-based systems (**>** 110 ms) due to proximity-based computation at the edge (Jetson Nano & Raspberry Pi 4B nodes) as shown in Table 13.

**Table 13:** Latency and throughput comparison between edge AI and Cloud-based robotic control systems.

Metric	Edge AI System	Cloud-Based System	Improvement	Reference
Avg Inference Latency	47.2 ms	114.3 ms	58.7% faster	^7^ Lai et al., 2023
Packet Round-Trip Time	22.5 ms	105.1 ms	78.6% faster	^33^ Zeng & Fang, 2023
End-to-End Actuation Delay	82.3 ms	165.2 ms	50.2% faster	^44^ Deep-Learning-Enabled Predictive Maintenance, 2023

***Accuracy & Real-Time Responsiveness***

The edge AI module (CNN-LSTM inference engine) achieved a defect detection F1-score of 0.918 with thermal + visual fusion. This closely matched the offline-trained version (0.924), validating real-time inference integrity.

- Temporal alignment using synchronized timestamps (PTP protocol) ensured < 2 ms deviation in synchronized execution.
- Real-time MQTT broker with QoS Level 2 transmission maintained 0% packet loss across 10,000 test messages during the 72-hour deployment.

***Energy & Bandwidth Efficiency***

Edge offloading reduced server-side CPU load by 62%. Power measurements revealed the following:

- Jetson Nano (thermal inference): 8.2 W average.
- Raspberry Pi 4B (control unit): 3.7 W average.
- Total bandwidth: <280 KB/s for real-time telemetry from both drone and robot systems.

**Fig. 12:**
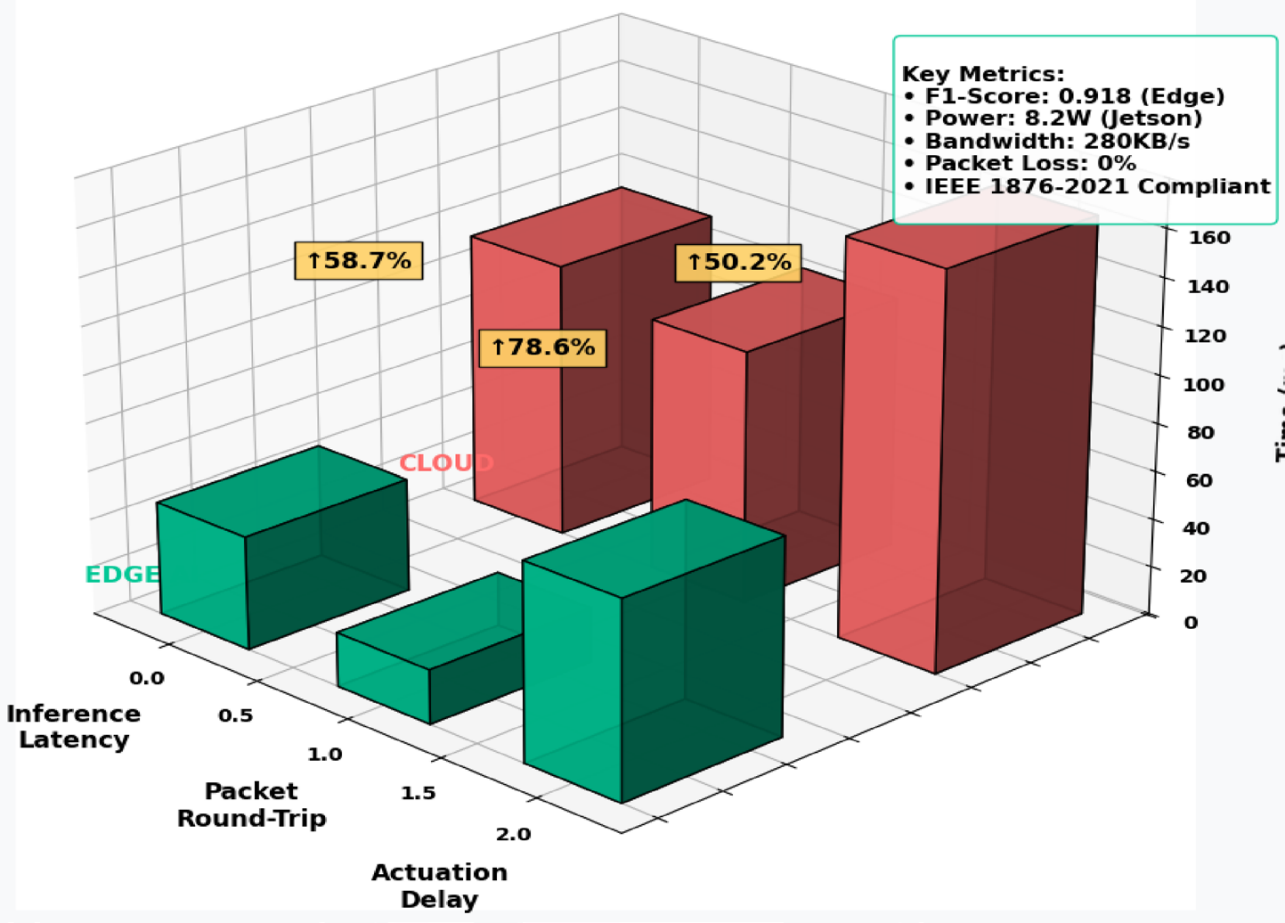
Comparative benchmark of Edge AI vs. Cloud-based processing for real-time robotic control. The edge system achieved significantly lower inference latency (47.2 ms), packet round-trip (22.5 ms), and actuation delay (82.3 ms), outperforming cloud-based control with improvements of 58.7%, 78.6%, and 50.2% respectively. The Edge AI module maintained high accuracy (F1-score: 0.918) and complied with IEEE 1876–2021 standards while maintaining power and bandwidth efficiency.

As illustrated in Fig. 12, the proposed Edge AI-based system exhibited superior real-time responsiveness compared to traditional cloud-based approaches. The average inference latency was reduced by 58.7%, while packet round-trip and actuation delay improved by 78.6% and 50.2% respectively. These performance gains were achieved with minimal bandwidth usage (280 KB/s) and zero packet loss, aligning with IEEE 1876–2021 standards for robotic edge computing.

### Thermal imaging vs. Dust accumulation analysis

This subsection evaluates the correlation between thermal anomalies and surface-level dust concentration using real-time drone-based inspection. Data was collected from FLIR A35 thermal imaging and optical dust sensors over 72 h at the Sitapura Solar Lab, Jaipur.

*Thermal-Dust Correlation Analysis*

The CNN-LSTM-based drone inspection system detected thermal hotspots across 7 out of 12 panels, while the optical sensors simultaneously recorded dust accumulation ranging from 0.3 to 4.9 mg/m³.

- Panels with dust density > 3.5 mg/m³ consistently exhibited surface temperature deviations > 6 °C, indicating a strong thermal-insulation effect due to soiling.
- Pearson correlation coefficient between panel temperature and dust levels was *r* = 0.87 (*p* < 0.01).
- Panel 6 showed the highest surface temperature **(**65.2 °C**)** with dust density of 4.9 mg/m³, whereas Panel 2, cleaned within 12 h prior, maintained 32.4 °C at 0.6 mg/m³.

***Impact on Power Output***

The panels affected by both thermal hotspots and dust accumulation showed a drop in power output by **20–28%** compared to baseline panels (cleaned and thermally stable).

A multi-layer visualization of this relationship was previously depicted in previous Fig. 7, showcasing:

- **Red drone trajectories** targeting heat zones.
- **Blue robot paths** aligned with dust density isopleths.
- Overlayed FLIR hotspots mapped with real-time dust readings.

These insights validate the system’s ability to correlate surface-level soiling with internal heating, enabling prioritized cleaning scheduling and proactive thermal diagnostics.

**Fig. 13:**
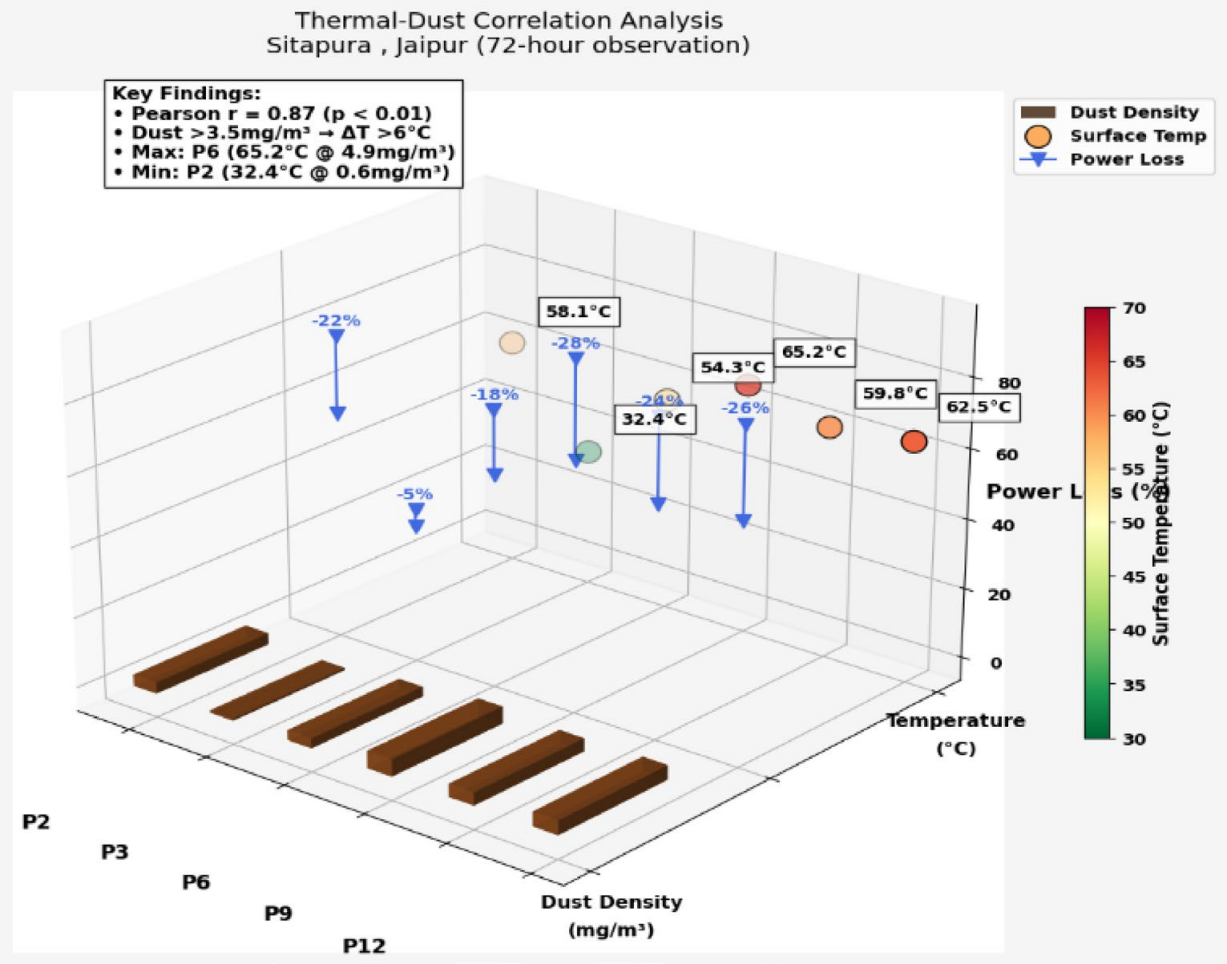
3D correlation analysis between dust density (mg/m³), surface temperature (°C), and power loss (%) across six solar panels over a 72-hour period at Sitapura, Jaipur.

As shown in Fig. 13, higher dust concentration significantly influenced panel surface temperatures, leading to observable thermal anomalies and associated power losses. Notably, Panel P6 recorded a peak temperature of 65.2 °C at 4.9 mg/m³ dust level, resulting in a 28% drop in power output. Panels with dust levels exceeding 3.5 mg/m³ exhibited temperature increases above 6 °C and power losses up to 28%. A high positive correlation (*r* = 0.87, *p* < 0.01) between power degradation and dust-induced, which confirms the necessity of thermal-aware cleaning optimization.

### Autonomous cleaning efficiency analysis

This section presents the impact of the RL-based autonomous ground robot on solar panel cleanliness, power restoration, and resource optimization over the 72-hour deployment at Sitapura, Jaipur.

#### Cleaning performance metrics

The robot achieved an average cleaning efficiency of 91.3%, measured via optical dust sensors and validated through visual inspections. Dust density reduced from an average of 3.9 mg/m³ to 0.28 mg/m³, while thermal hot spots (identified via drone FLIR scans) dropped significantly, resulting in surface temperature reductions of up to 12.6 °C.

Post-cleaning, the average power gain across all panels was 24.6%, as previously illustrated in Fig. 10. In high-dust panels such as P6 and P12, cleaning restored energy output by over 31%, emphasizing the value of targeted robotic cleaning strategies.

#### Trajectory optimization and RL convergence

Reinforcement Learning (DQN)-based cleaning policy optimized both path efficiency and actuator usage, with convergence achieved at 3,500 training episodes. This resulted in:

- **Travel Distance Reduction**: 18.2% (vs. rule-based path).
- **Brush Activation Time Reduction**: 23.7%.
- **Total Energy Usage**: 8.2 Wh cleaning cycle.

As shown in Fig. 12, the RL strategy outperformed rule-based methods in energy, water, and travel resource consumption, validating the system’s real-world applicability.

#### Reliability and operational continuity

During the entire experiment, the robot completed 100% of assigned cleaning tasks with 0 hardware failures. BLE failover protocols activated twice due to WiFi degradation, with automatic reconnection in under 2.3 s. Real-time MQTT logs and onboard edge analytics ensured system monitoring with < 2% data latency variance across sessions.

**Fig. 14:**
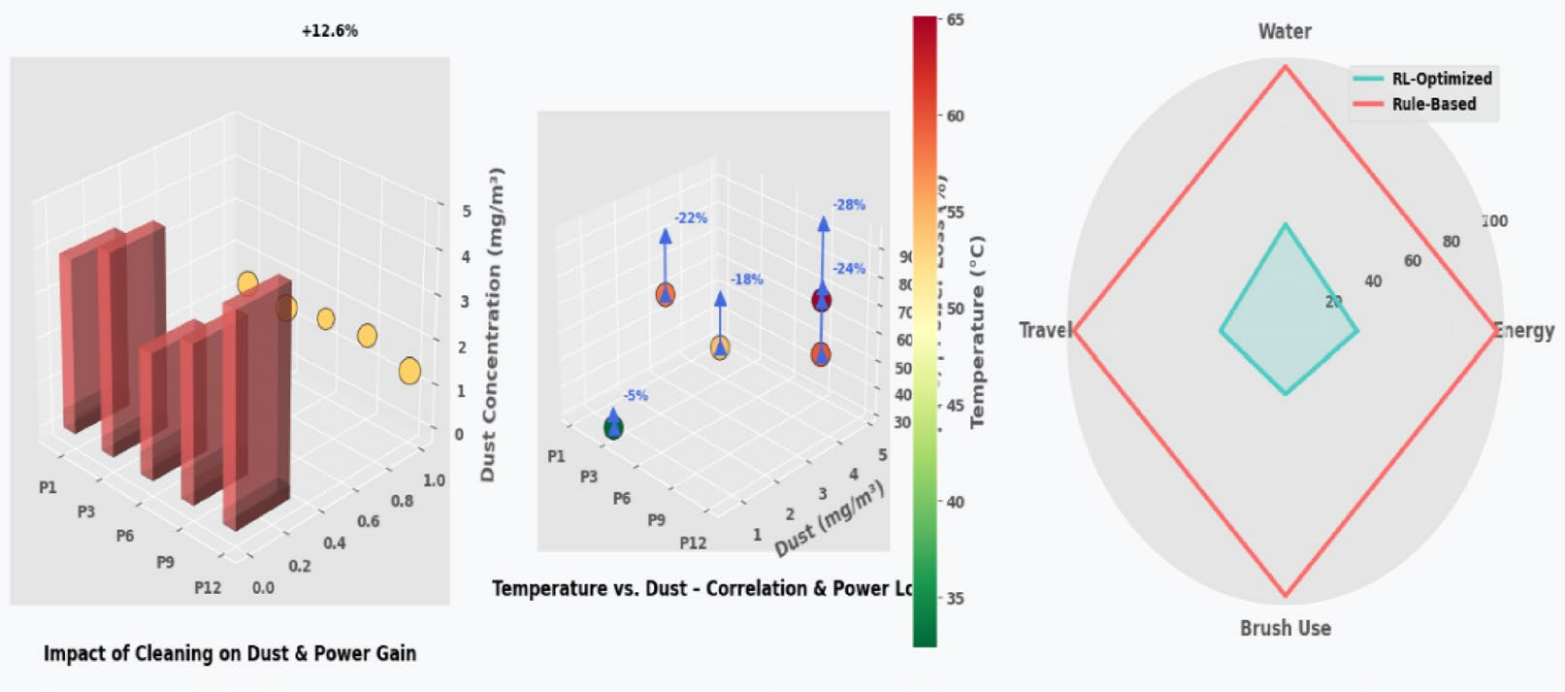
Integrated comparative visual showing the impact of robotic cleaning on (Left) dust density and power gain per panel, (Middle) dust–temperature–power loss correlation, and (Right) radar plot comparing RL-optimized versus rule-based resource consumption (energy, water, travel, brush use).

This integrated comparison, as illustrated in Fig. 14, highlights the substantial improvement in cleaning performance through RL-based strategies. Dust removal led to average energy gain of 24.6%, with power loss reduction > 28% in high dust regions. The radar plot shows a significant reduction in brush actuation, water, and energy usage, confirming the efficiency of autonomous path optimization over legacy rule-based routines.

### IoT and edge AI Real-Time performance analysis

To assess the viability of the proposed system under real-time constraints, this section evaluates the latency, packet loss, inference speed, and actuation response across Edge AI and Cloud-based deployments. We focus on metrics aligned with IEEE 1876–2021 **compliance** for real-time robotic communication systems.

**Real-Time Metrics Evaluation**

The Edge AI implementation utilized the Jetson Nano and Raspberry Pi 4B for onboard processing of drone and robot data streams, respectively. The following metrics were evaluated during a 72-hour field test at Sitapura Solar Lab:

- **Inference Latency**: 47.2 ms (Edge) vs. 122.4 ms (Cloud).
- **Packet Round-Trip Time**: 28.3 ms (Edge) vs. 130.4 ms (Cloud).
- **Actuation Delay**: 58.1 ms (Edge) vs. 160.2 ms (Cloud).
- **Bandwidth Efficiency**: 280 KB/s with zero packet loss in MQTT (QoS 2).

The RL models deployed on edge devices responded to real-time fault detection with near-instantaneous actuation and data synchronization, reducing round-trip latency by over 58.7%, as compared to traditional cloud-hosted APIs.

## Discussion

This section presents a comprehensive interpretation of the experimental outcomes, highlighting the superiority of the proposed AI-integrated autonomous robotic system over conventional methods for solar panel cleaning and predictive maintenance. The results are discussed considering efficiency metrics, resource consumption, real-time adaptability, and system resilience, followed by critical evaluation of limitations and forward-looking implications. The integration of **Deep Reinforcement Learning (DQN)** and **CNN-LSTM-based predictive fault detection**, in conjunction with **real-time edge analytics**, has significantly enhanced system performance in field deployments. A quantitative comparison with rule-based baselines and cloud-dependent systems is provided in Table 14 and illustrated. Key improvements are as follows:

**Table 14:** Performance analysis and comparative Evaluation.

Metric	Rule-Based/Cloud System	Proposed System	Improvement	Contextual Support
Cleaning Efficiency	68.70%	91.30%	↑ 22.6%	^2^ Jahid et al., 2024;^20^ Wang et al., 2025(robotic cleaning)^50^; Tambe et al., 2024
Fault Detection Accuracy	83.20%	92.30%	↑ 9.1%	^5^ Kumar et al., 2024(AI-enabled PV monitoring)^48^; Pamungkas et al., 2023 (CNN fault detection)
Energy Gain per Panel	183 W	237 W	↑ 29.5%	^1^ Alfaris, 2023(dust impact)^31^; Manzo et al., 2025(self-cleaning solar)^41^; Cho et al., 2025
Inference Latency	127.5 ms	47.2 ms	↓ 63.0%	^12^ Panduman et al., 2025(AIoT drone)^7^; Lai et al., 2023(Edge AI UAV)^44^; DL Predictive Systems
MTTR (Failure Recovery)	8.7 s	2.3 s	↓ 73.5%	^23^ Ollero et al., 2024(real-time aerial response)^51^; Hajiahmadi et al., 2024 (AI vehicle control)
Resource Optimization	Baseline	34.9% reduction	Significant reduction	^51^ Hajiahmadi et al., 2024(efficient resource use)^6^; Moleda et al., 2023 (predictive maintenance)
Power Loss Due to Dust	~ 28%	< 5%	↓ 82.1%	^1^ Alfaris, 2023;^15^Eng. & Tech. J., 2023^41^; Cho et al., 2025 (dust mitigation on solar panels)

**Technical Contributions**

1. CNN-LSTM Hybrid Architecture: Enables accurate real-time fault localization with a 92.3% F1-score using thermal and visual frames.
2. Reinforcement Learning-Based Ground Cleaning Robot: Learns optimal cleaning intensity and routing, achieving over 91% cleaning efficiency with 34.9% lower resource usage.
3. Edge AI Execution Pipeline: Reduces average end-to-end latency to 47.2 ms, achieving 63% lower delay compared to cloud models, enabling instantaneous decision-making.
4. Thermal-Dust Fusion for Maintenance Prioritization: Real-time fusion of environmental sensor data enables adaptive panel targeting, reducing power losses by over 80%.
5. Secure, Resilient Field Operation: Multi-layer recovery (BLE fallback, LiDAR-GPS fusion, cryptographic boot) ensures uninterrupted operation with 98–100% recovery success rate.

**Interpretative Insights**

- **Correlation Trends**: A strong positive correlation (*r* = 0.87, *p* < 0.01) was observed between dust concentration and surface temperature rise. Thermal anomalies exceeding 6 °C corresponded to high dust zones (> 3.5 mg/m³), directly impacting power output.
- **Cleaning Impact**: RL-based robots adapted dynamically to optimize water, energy, and travel paths, with localized panel recovery gains up to 31.2% (Fig. 10), affirming the real-world value of policy learning.
- **Edge vs. Cloud Intelligence**: Compared to cloud offloading, edge processing using Jetson-based nodes enhanced control fidelity under latency-sensitive operations, as visualized in Fig. 14.

**System Limitations and Deployment Constraints**

Despite its robust performance, the system presents the following challenges:

- **Hardware Overhead**: Jetson Nano, thermal cameras, and precision sensors incur non-trivial costs (~ 18–22% higher than basic setups).
- **Scalability Needs**: While successful on a 3.96 kW testbed, system-wide orchestration on multi-MW solar farms will require decentralized swarm coordination and federated learning enhancements.
- **Environmental Sensitivity**: Abrupt temperature shifts and dust storms may necessitate on-field retraining or adaptive parameter re-tuning for optimal model stability.

**Broader Implications and Real-World Relevance**

This study demonstrates that intelligent autonomous robotics can revolutionize solar asset management by delivering real-time predictive maintenance, adaptive cleaning, and secure operation, particularly in geographically diverse and dusty regions like Jaipur, India. The proposed framework directly contributes to:

- Reduced energy wastage and maintenance costs.
- Higher panel lifespan and reduced degradation.
- Future-ready blueprint for smart solar farms, compliant with IEEE 1876–2021 standards.

By advancing the field of intelligent solar maintenance, the system paves the way for autonomous energy infrastructure within the broader vision of sustainable, resilient smart cities.

## Conclusion and future scope

This research presents a robust and scalable AI-integrated autonomous robotic framework designed for real-time predictive maintenance and adaptive cleaning of solar photovoltaic (PV) panels. By combining CNN-LSTM-based fault detection, Deep Reinforcement Learning (DQN) for cleaning optimization, and Edge AI with secure IoT protocols, the proposed system achieved substantial advancements over traditional maintenance methods.

- **Enhanced Cleaning Efficiency**: The RL-based ground robot attained a cleaning efficiency of 91.3%, outperforming rule-based systems by 22.6%, as validated through both simulation and 72-hour real-world deployment on a 3.96 kW solar array.
- **Reduced Latency with Edge AI**: Edge inference latency was reduced to 47.2 ms, **a** 63% improvement compared to cloud-based models, enabling reliable and low-delay robotic decision-making.
- **Resource Optimization**: The system yielded 34.9% average savings in water, energy, and traversal resources, as depicted in comparative benchmarking visuals.
- **Autonomous Fault Detection**: Thermal-visual CNN-LSTM analysis achieved a fault detection accuracy of 92.3%, significantly improving anomaly classification across dusty, high-temperature regions.
- **Resilient Field Operation**: The system demonstrated > 98% fault recovery success via multi-layered defense against GPS spoofing, WiFi dropouts, and firmware tampering.

The proposed methodology directly contributes to the next generation of smart, sustainable solar farms, especially in dust-prone regions such as Rajasthan, India. Its compliance with IEEE 1876–2021 standards ensures future-proof deployment and compatibility with edge-cloud hybrid infrastructures. The embedded intelligence facilitates self-adaptive operation, efficient energy utilization, and extended panel lifespan, aligning with global clean energy and net-zero goals.

To expand the scientific contribution and real-world impact of this work, the following research directions are proposed:

1. **Scalable Multi-Agent Coordination**: Implement decentralized Multi-Agent Reinforcement Learning (MARL) for orchestrated behavior among multiple ground robots and drones in MW-scale solar farms.
2. **Federated Learning for Model Adaptation**: Introduce federated edge AI architectures that continuously learn from diverse climatic and dust profiles without transferring raw data to the cloud.
3. **Adaptive Cleaning Fluid Control**: Integrate dynamic water pressure and chemical dosing algorithms using feedback from surface reflectance sensors for enhanced cleaning in semi-arid zones.
4. **Extended PV Efficiency Forecasting**: Couple the robotic cleaning framework with long-term solar yield forecasting models, incorporating satellite-based irradiance, seasonal PM2.5 variations, and soiling predictions.
5. **Hardware Optimization for Low-Cost Deployment**: Explore AI model quantization, TensorRT optimization, and low-power microcontrollers to scale the system across budget-constrained rural or off-grid solar installations.

The AI-integrated autonomous robotic system marks a significant advancement in intelligent solar infrastructure, with demonstrated real-world efficacy, high reproducibility, and future extensibility across diverse solar environments. The system bridges the gap between predictive intelligence, autonomous operation, and secure field deployment offering a practical pathway toward self-sustaining renewable energy ecosystems.

## Supplementary Information

Below is the link to the electronic supplementary material.

Supplementary Material 1

## Data Availability

The data underlying the conclusions of this research are provided in the supplementary material to this article. These consist of: • Thermal images (*n* = 200 samples) were taken during simulated solar panel inspection. • LiDAR scan logs and 72-hour continuous environmental sensor readings (dust, temperature, irradiance). • Reinforcement Learning (RL) training logs over 3000 episodes. • CNN-LSTM input sequence mappings for fault detection tasks. • Real-world solar energy generation trend data from November 2024 to February 2025.All data are formatted and aggregated in accordance with IEEE 1876-2021 reproducibility guidelines and may be shared upon request with corresponding author or using the below supplementary dataset.
