# Supplementary figures and images for: AI-Integrated autonomous robotics for solar panel cleaning and predictive maintenance using drone and ground-based systems (part 2 of 2)
Source: Sci Rep. 2025 Sep 1;15:32187. doi: 10.1038/s41598-025-17313-6 (PMC12402262; doi:10.1038/s41598-025-17313-6)

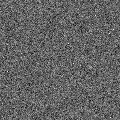

Supplement: Supplementary file 1 — Supplementary Material 1 [file 41598_2025_17313_MOESM1_ESM.zip › thermal_images/thermal_frame_0101.png]

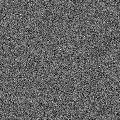

Supplement: Supplementary file 1 — Supplementary Material 1 [file 41598_2025_17313_MOESM1_ESM.zip › thermal_images/thermal_frame_0102.png]

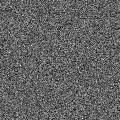

Supplement: Supplementary file 1 — Supplementary Material 1 [file 41598_2025_17313_MOESM1_ESM.zip › thermal_images/thermal_frame_0103.png]

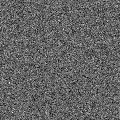

Supplement: Supplementary file 1 — Supplementary Material 1 [file 41598_2025_17313_MOESM1_ESM.zip › thermal_images/thermal_frame_0104.png]

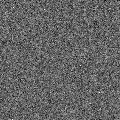

Supplement: Supplementary file 1 — Supplementary Material 1 [file 41598_2025_17313_MOESM1_ESM.zip › thermal_images/thermal_frame_0105.png]

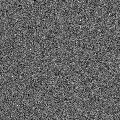

Supplement: Supplementary file 1 — Supplementary Material 1 [file 41598_2025_17313_MOESM1_ESM.zip › thermal_images/thermal_frame_0106.png]

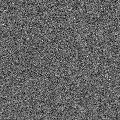

Supplement: Supplementary file 1 — Supplementary Material 1 [file 41598_2025_17313_MOESM1_ESM.zip › thermal_images/thermal_frame_0107.png]

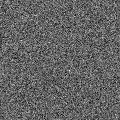

Supplement: Supplementary file 1 — Supplementary Material 1 [file 41598_2025_17313_MOESM1_ESM.zip › thermal_images/thermal_frame_0108.png]

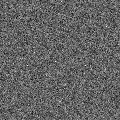

Supplement: Supplementary file 1 — Supplementary Material 1 [file 41598_2025_17313_MOESM1_ESM.zip › thermal_images/thermal_frame_0109.png]

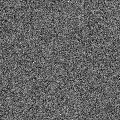

Supplement: Supplementary file 1 — Supplementary Material 1 [file 41598_2025_17313_MOESM1_ESM.zip › thermal_images/thermal_frame_0110.png]

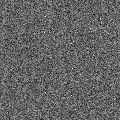

Supplement: Supplementary file 1 — Supplementary Material 1 [file 41598_2025_17313_MOESM1_ESM.zip › thermal_images/thermal_frame_0111.png]

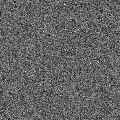

Supplement: Supplementary file 1 — Supplementary Material 1 [file 41598_2025_17313_MOESM1_ESM.zip › thermal_images/thermal_frame_0112.png]

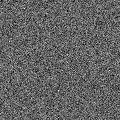

Supplement: Supplementary file 1 — Supplementary Material 1 [file 41598_2025_17313_MOESM1_ESM.zip › thermal_images/thermal_frame_0113.png]

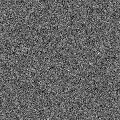

Supplement: Supplementary file 1 — Supplementary Material 1 [file 41598_2025_17313_MOESM1_ESM.zip › thermal_images/thermal_frame_0114.png]

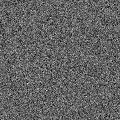

Supplement: Supplementary file 1 — Supplementary Material 1 [file 41598_2025_17313_MOESM1_ESM.zip › thermal_images/thermal_frame_0115.png]

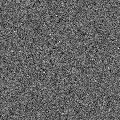

Supplement: Supplementary file 1 — Supplementary Material 1 [file 41598_2025_17313_MOESM1_ESM.zip › thermal_images/thermal_frame_0116.png]

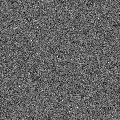

Supplement: Supplementary file 1 — Supplementary Material 1 [file 41598_2025_17313_MOESM1_ESM.zip › thermal_images/thermal_frame_0117.png]

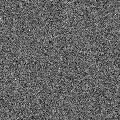

Supplement: Supplementary file 1 — Supplementary Material 1 [file 41598_2025_17313_MOESM1_ESM.zip › thermal_images/thermal_frame_0118.png]

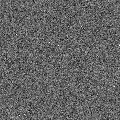

Supplement: Supplementary file 1 — Supplementary Material 1 [file 41598_2025_17313_MOESM1_ESM.zip › thermal_images/thermal_frame_0119.png]

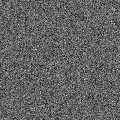

Supplement: Supplementary file 1 — Supplementary Material 1 [file 41598_2025_17313_MOESM1_ESM.zip › thermal_images/thermal_frame_0120.png]

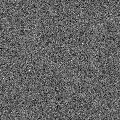

Supplement: Supplementary file 1 — Supplementary Material 1 [file 41598_2025_17313_MOESM1_ESM.zip › thermal_images/thermal_frame_0121.png]

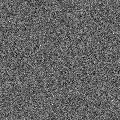

Supplement: Supplementary file 1 — Supplementary Material 1 [file 41598_2025_17313_MOESM1_ESM.zip › thermal_images/thermal_frame_0122.png]

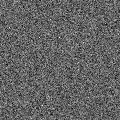

Supplement: Supplementary file 1 — Supplementary Material 1 [file 41598_2025_17313_MOESM1_ESM.zip › thermal_images/thermal_frame_0123.png]

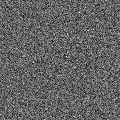

Supplement: Supplementary file 1 — Supplementary Material 1 [file 41598_2025_17313_MOESM1_ESM.zip › thermal_images/thermal_frame_0124.png]

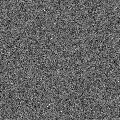

Supplement: Supplementary file 1 — Supplementary Material 1 [file 41598_2025_17313_MOESM1_ESM.zip › thermal_images/thermal_frame_0125.png]

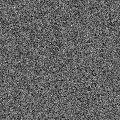

Supplement: Supplementary file 1 — Supplementary Material 1 [file 41598_2025_17313_MOESM1_ESM.zip › thermal_images/thermal_frame_0126.png]

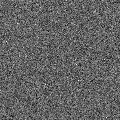

Supplement: Supplementary file 1 — Supplementary Material 1 [file 41598_2025_17313_MOESM1_ESM.zip › thermal_images/thermal_frame_0127.png]

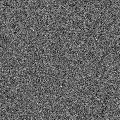

Supplement: Supplementary file 1 — Supplementary Material 1 [file 41598_2025_17313_MOESM1_ESM.zip › thermal_images/thermal_frame_0128.png]

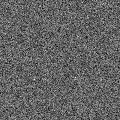

Supplement: Supplementary file 1 — Supplementary Material 1 [file 41598_2025_17313_MOESM1_ESM.zip › thermal_images/thermal_frame_0129.png]

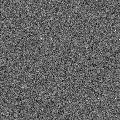

Supplement: Supplementary file 1 — Supplementary Material 1 [file 41598_2025_17313_MOESM1_ESM.zip › thermal_images/thermal_frame_0130.png]

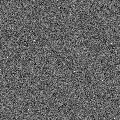

Supplement: Supplementary file 1 — Supplementary Material 1 [file 41598_2025_17313_MOESM1_ESM.zip › thermal_images/thermal_frame_0131.png]

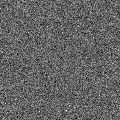

Supplement: Supplementary file 1 — Supplementary Material 1 [file 41598_2025_17313_MOESM1_ESM.zip › thermal_images/thermal_frame_0132.png]

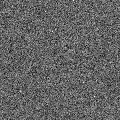

Supplement: Supplementary file 1 — Supplementary Material 1 [file 41598_2025_17313_MOESM1_ESM.zip › thermal_images/thermal_frame_0133.png]

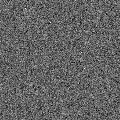

Supplement: Supplementary file 1 — Supplementary Material 1 [file 41598_2025_17313_MOESM1_ESM.zip › thermal_images/thermal_frame_0134.png]

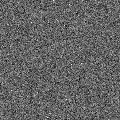

Supplement: Supplementary file 1 — Supplementary Material 1 [file 41598_2025_17313_MOESM1_ESM.zip › thermal_images/thermal_frame_0135.png]

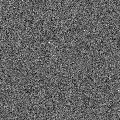

Supplement: Supplementary file 1 — Supplementary Material 1 [file 41598_2025_17313_MOESM1_ESM.zip › thermal_images/thermal_frame_0136.png]

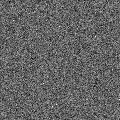

Supplement: Supplementary file 1 — Supplementary Material 1 [file 41598_2025_17313_MOESM1_ESM.zip › thermal_images/thermal_frame_0137.png]

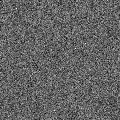

Supplement: Supplementary file 1 — Supplementary Material 1 [file 41598_2025_17313_MOESM1_ESM.zip › thermal_images/thermal_frame_0138.png]

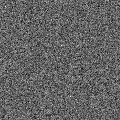

Supplement: Supplementary file 1 — Supplementary Material 1 [file 41598_2025_17313_MOESM1_ESM.zip › thermal_images/thermal_frame_0139.png]

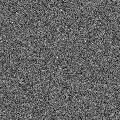

Supplement: Supplementary file 1 — Supplementary Material 1 [file 41598_2025_17313_MOESM1_ESM.zip › thermal_images/thermal_frame_0140.png]

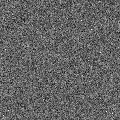

Supplement: Supplementary file 1 — Supplementary Material 1 [file 41598_2025_17313_MOESM1_ESM.zip › thermal_images/thermal_frame_0141.png]

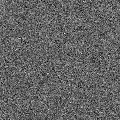

Supplement: Supplementary file 1 — Supplementary Material 1 [file 41598_2025_17313_MOESM1_ESM.zip › thermal_images/thermal_frame_0142.png]

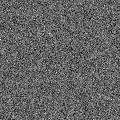

Supplement: Supplementary file 1 — Supplementary Material 1 [file 41598_2025_17313_MOESM1_ESM.zip › thermal_images/thermal_frame_0143.png]

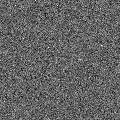

Supplement: Supplementary file 1 — Supplementary Material 1 [file 41598_2025_17313_MOESM1_ESM.zip › thermal_images/thermal_frame_0144.png]

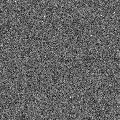

Supplement: Supplementary file 1 — Supplementary Material 1 [file 41598_2025_17313_MOESM1_ESM.zip › thermal_images/thermal_frame_0145.png]

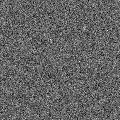

Supplement: Supplementary file 1 — Supplementary Material 1 [file 41598_2025_17313_MOESM1_ESM.zip › thermal_images/thermal_frame_0146.png]

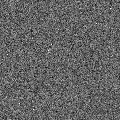

Supplement: Supplementary file 1 — Supplementary Material 1 [file 41598_2025_17313_MOESM1_ESM.zip › thermal_images/thermal_frame_0147.png]

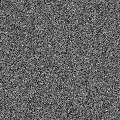

Supplement: Supplementary file 1 — Supplementary Material 1 [file 41598_2025_17313_MOESM1_ESM.zip › thermal_images/thermal_frame_0148.png]

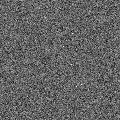

Supplement: Supplementary file 1 — Supplementary Material 1 [file 41598_2025_17313_MOESM1_ESM.zip › thermal_images/thermal_frame_0149.png]

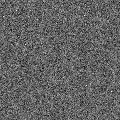

Supplement: Supplementary file 1 — Supplementary Material 1 [file 41598_2025_17313_MOESM1_ESM.zip › thermal_images/thermal_frame_0150.png]

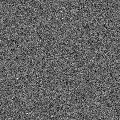

Supplement: Supplementary file 1 — Supplementary Material 1 [file 41598_2025_17313_MOESM1_ESM.zip › thermal_images/thermal_frame_0151.png]

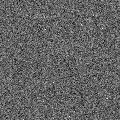

Supplement: Supplementary file 1 — Supplementary Material 1 [file 41598_2025_17313_MOESM1_ESM.zip › thermal_images/thermal_frame_0152.png]

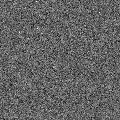

Supplement: Supplementary file 1 — Supplementary Material 1 [file 41598_2025_17313_MOESM1_ESM.zip › thermal_images/thermal_frame_0153.png]

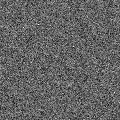

Supplement: Supplementary file 1 — Supplementary Material 1 [file 41598_2025_17313_MOESM1_ESM.zip › thermal_images/thermal_frame_0154.png]

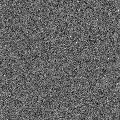

Supplement: Supplementary file 1 — Supplementary Material 1 [file 41598_2025_17313_MOESM1_ESM.zip › thermal_images/thermal_frame_0155.png]

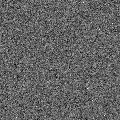

Supplement: Supplementary file 1 — Supplementary Material 1 [file 41598_2025_17313_MOESM1_ESM.zip › thermal_images/thermal_frame_0156.png]

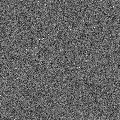

Supplement: Supplementary file 1 — Supplementary Material 1 [file 41598_2025_17313_MOESM1_ESM.zip › thermal_images/thermal_frame_0157.png]

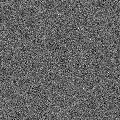

Supplement: Supplementary file 1 — Supplementary Material 1 [file 41598_2025_17313_MOESM1_ESM.zip › thermal_images/thermal_frame_0158.png]

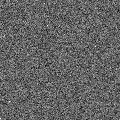

Supplement: Supplementary file 1 — Supplementary Material 1 [file 41598_2025_17313_MOESM1_ESM.zip › thermal_images/thermal_frame_0159.png]

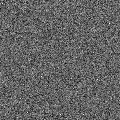

Supplement: Supplementary file 1 — Supplementary Material 1 [file 41598_2025_17313_MOESM1_ESM.zip › thermal_images/thermal_frame_0160.png]

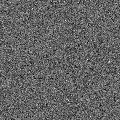

Supplement: Supplementary file 1 — Supplementary Material 1 [file 41598_2025_17313_MOESM1_ESM.zip › thermal_images/thermal_frame_0161.png]

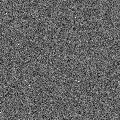

Supplement: Supplementary file 1 — Supplementary Material 1 [file 41598_2025_17313_MOESM1_ESM.zip › thermal_images/thermal_frame_0162.png]

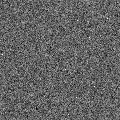

Supplement: Supplementary file 1 — Supplementary Material 1 [file 41598_2025_17313_MOESM1_ESM.zip › thermal_images/thermal_frame_0163.png]

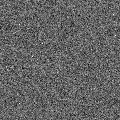

Supplement: Supplementary file 1 — Supplementary Material 1 [file 41598_2025_17313_MOESM1_ESM.zip › thermal_images/thermal_frame_0164.png]

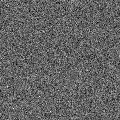

Supplement: Supplementary file 1 — Supplementary Material 1 [file 41598_2025_17313_MOESM1_ESM.zip › thermal_images/thermal_frame_0165.png]

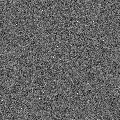

Supplement: Supplementary file 1 — Supplementary Material 1 [file 41598_2025_17313_MOESM1_ESM.zip › thermal_images/thermal_frame_0166.png]

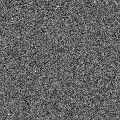

Supplement: Supplementary file 1 — Supplementary Material 1 [file 41598_2025_17313_MOESM1_ESM.zip › thermal_images/thermal_frame_0167.png]

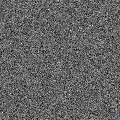

Supplement: Supplementary file 1 — Supplementary Material 1 [file 41598_2025_17313_MOESM1_ESM.zip › thermal_images/thermal_frame_0168.png]

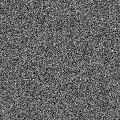

Supplement: Supplementary file 1 — Supplementary Material 1 [file 41598_2025_17313_MOESM1_ESM.zip › thermal_images/thermal_frame_0169.png]

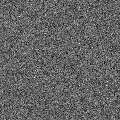

Supplement: Supplementary file 1 — Supplementary Material 1 [file 41598_2025_17313_MOESM1_ESM.zip › thermal_images/thermal_frame_0170.png]

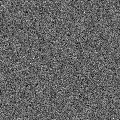

Supplement: Supplementary file 1 — Supplementary Material 1 [file 41598_2025_17313_MOESM1_ESM.zip › thermal_images/thermal_frame_0171.png]

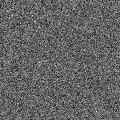

Supplement: Supplementary file 1 — Supplementary Material 1 [file 41598_2025_17313_MOESM1_ESM.zip › thermal_images/thermal_frame_0172.png]

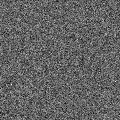

Supplement: Supplementary file 1 — Supplementary Material 1 [file 41598_2025_17313_MOESM1_ESM.zip › thermal_images/thermal_frame_0173.png]

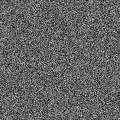

Supplement: Supplementary file 1 — Supplementary Material 1 [file 41598_2025_17313_MOESM1_ESM.zip › thermal_images/thermal_frame_0174.png]

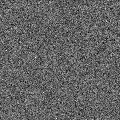

Supplement: Supplementary file 1 — Supplementary Material 1 [file 41598_2025_17313_MOESM1_ESM.zip › thermal_images/thermal_frame_0175.png]

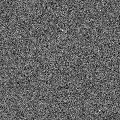

Supplement: Supplementary file 1 — Supplementary Material 1 [file 41598_2025_17313_MOESM1_ESM.zip › thermal_images/thermal_frame_0176.png]

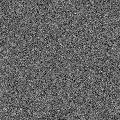

Supplement: Supplementary file 1 — Supplementary Material 1 [file 41598_2025_17313_MOESM1_ESM.zip › thermal_images/thermal_frame_0177.png]

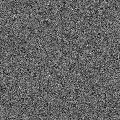

Supplement: Supplementary file 1 — Supplementary Material 1 [file 41598_2025_17313_MOESM1_ESM.zip › thermal_images/thermal_frame_0178.png]

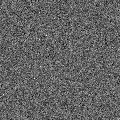

Supplement: Supplementary file 1 — Supplementary Material 1 [file 41598_2025_17313_MOESM1_ESM.zip › thermal_images/thermal_frame_0179.png]

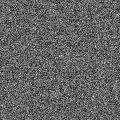

Supplement: Supplementary file 1 — Supplementary Material 1 [file 41598_2025_17313_MOESM1_ESM.zip › thermal_images/thermal_frame_0180.png]

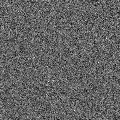

Supplement: Supplementary file 1 — Supplementary Material 1 [file 41598_2025_17313_MOESM1_ESM.zip › thermal_images/thermal_frame_0181.png]

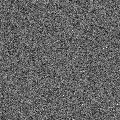

Supplement: Supplementary file 1 — Supplementary Material 1 [file 41598_2025_17313_MOESM1_ESM.zip › thermal_images/thermal_frame_0182.png]

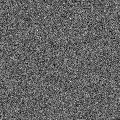

Supplement: Supplementary file 1 — Supplementary Material 1 [file 41598_2025_17313_MOESM1_ESM.zip › thermal_images/thermal_frame_0183.png]

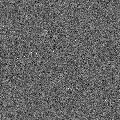

Supplement: Supplementary file 1 — Supplementary Material 1 [file 41598_2025_17313_MOESM1_ESM.zip › thermal_images/thermal_frame_0184.png]

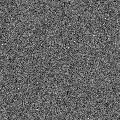

Supplement: Supplementary file 1 — Supplementary Material 1 [file 41598_2025_17313_MOESM1_ESM.zip › thermal_images/thermal_frame_0185.png]

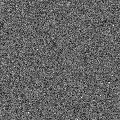

Supplement: Supplementary file 1 — Supplementary Material 1 [file 41598_2025_17313_MOESM1_ESM.zip › thermal_images/thermal_frame_0186.png]

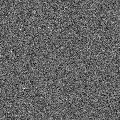

Supplement: Supplementary file 1 — Supplementary Material 1 [file 41598_2025_17313_MOESM1_ESM.zip › thermal_images/thermal_frame_0187.png]

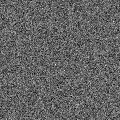

Supplement: Supplementary file 1 — Supplementary Material 1 [file 41598_2025_17313_MOESM1_ESM.zip › thermal_images/thermal_frame_0188.png]

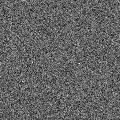

Supplement: Supplementary file 1 — Supplementary Material 1 [file 41598_2025_17313_MOESM1_ESM.zip › thermal_images/thermal_frame_0189.png]

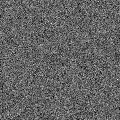

Supplement: Supplementary file 1 — Supplementary Material 1 [file 41598_2025_17313_MOESM1_ESM.zip › thermal_images/thermal_frame_0190.png]

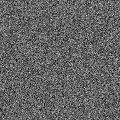

Supplement: Supplementary file 1 — Supplementary Material 1 [file 41598_2025_17313_MOESM1_ESM.zip › thermal_images/thermal_frame_0191.png]

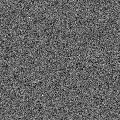

Supplement: Supplementary file 1 — Supplementary Material 1 [file 41598_2025_17313_MOESM1_ESM.zip › thermal_images/thermal_frame_0192.png]

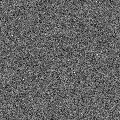

Supplement: Supplementary file 1 — Supplementary Material 1 [file 41598_2025_17313_MOESM1_ESM.zip › thermal_images/thermal_frame_0193.png]

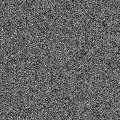

Supplement: Supplementary file 1 — Supplementary Material 1 [file 41598_2025_17313_MOESM1_ESM.zip › thermal_images/thermal_frame_0194.png]

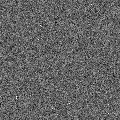

Supplement: Supplementary file 1 — Supplementary Material 1 [file 41598_2025_17313_MOESM1_ESM.zip › thermal_images/thermal_frame_0195.png]

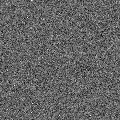

Supplement: Supplementary file 1 — Supplementary Material 1 [file 41598_2025_17313_MOESM1_ESM.zip › thermal_images/thermal_frame_0196.png]

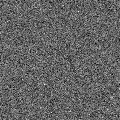

Supplement: Supplementary file 1 — Supplementary Material 1 [file 41598_2025_17313_MOESM1_ESM.zip › thermal_images/thermal_frame_0197.png]

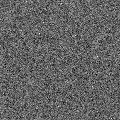

Supplement: Supplementary file 1 — Supplementary Material 1 [file 41598_2025_17313_MOESM1_ESM.zip › thermal_images/thermal_frame_0198.png]

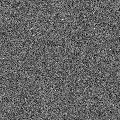

Supplement: Supplementary file 1 — Supplementary Material 1 [file 41598_2025_17313_MOESM1_ESM.zip › thermal_images/thermal_frame_0199.png]
