# Supplementary figures and images for: AI-Integrated autonomous robotics for solar panel cleaning and predictive maintenance using drone and ground-based systems (part 1 of 2)
Source: Sci Rep. 2025 Sep 1;15:32187. doi: 10.1038/s41598-025-17313-6 (PMC12402262; doi:10.1038/s41598-025-17313-6)

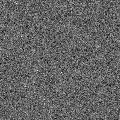

Supplement: Supplementary file 1 — Supplementary Material 1 [file 41598_2025_17313_MOESM1_ESM.zip › thermal_images/thermal_frame_0001.png]

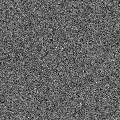

Supplement: Supplementary file 1 — Supplementary Material 1 [file 41598_2025_17313_MOESM1_ESM.zip › thermal_images/thermal_frame_0002.png]

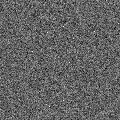

Supplement: Supplementary file 1 — Supplementary Material 1 [file 41598_2025_17313_MOESM1_ESM.zip › thermal_images/thermal_frame_0003.png]

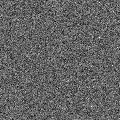

Supplement: Supplementary file 1 — Supplementary Material 1 [file 41598_2025_17313_MOESM1_ESM.zip › thermal_images/thermal_frame_0004.png]

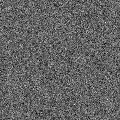

Supplement: Supplementary file 1 — Supplementary Material 1 [file 41598_2025_17313_MOESM1_ESM.zip › thermal_images/thermal_frame_0005.png]

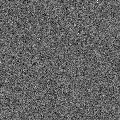

Supplement: Supplementary file 1 — Supplementary Material 1 [file 41598_2025_17313_MOESM1_ESM.zip › thermal_images/thermal_frame_0006.png]

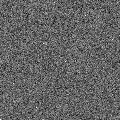

Supplement: Supplementary file 1 — Supplementary Material 1 [file 41598_2025_17313_MOESM1_ESM.zip › thermal_images/thermal_frame_0007.png]

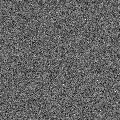

Supplement: Supplementary file 1 — Supplementary Material 1 [file 41598_2025_17313_MOESM1_ESM.zip › thermal_images/thermal_frame_0008.png]

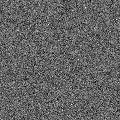

Supplement: Supplementary file 1 — Supplementary Material 1 [file 41598_2025_17313_MOESM1_ESM.zip › thermal_images/thermal_frame_0009.png]

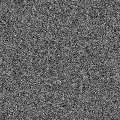

Supplement: Supplementary file 1 — Supplementary Material 1 [file 41598_2025_17313_MOESM1_ESM.zip › thermal_images/thermal_frame_0010.png]

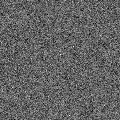

Supplement: Supplementary file 1 — Supplementary Material 1 [file 41598_2025_17313_MOESM1_ESM.zip › thermal_images/thermal_frame_0011.png]

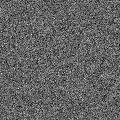

Supplement: Supplementary file 1 — Supplementary Material 1 [file 41598_2025_17313_MOESM1_ESM.zip › thermal_images/thermal_frame_0012.png]

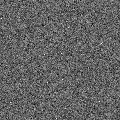

Supplement: Supplementary file 1 — Supplementary Material 1 [file 41598_2025_17313_MOESM1_ESM.zip › thermal_images/thermal_frame_0013.png]

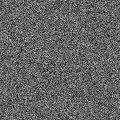

Supplement: Supplementary file 1 — Supplementary Material 1 [file 41598_2025_17313_MOESM1_ESM.zip › thermal_images/thermal_frame_0014.png]

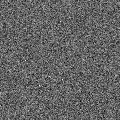

Supplement: Supplementary file 1 — Supplementary Material 1 [file 41598_2025_17313_MOESM1_ESM.zip › thermal_images/thermal_frame_0015.png]

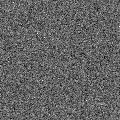

Supplement: Supplementary file 1 — Supplementary Material 1 [file 41598_2025_17313_MOESM1_ESM.zip › thermal_images/thermal_frame_0016.png]

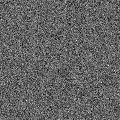

Supplement: Supplementary file 1 — Supplementary Material 1 [file 41598_2025_17313_MOESM1_ESM.zip › thermal_images/thermal_frame_0017.png]

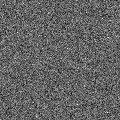

Supplement: Supplementary file 1 — Supplementary Material 1 [file 41598_2025_17313_MOESM1_ESM.zip › thermal_images/thermal_frame_0018.png]

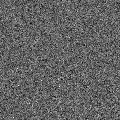

Supplement: Supplementary file 1 — Supplementary Material 1 [file 41598_2025_17313_MOESM1_ESM.zip › thermal_images/thermal_frame_0019.png]

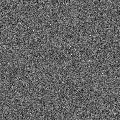

Supplement: Supplementary file 1 — Supplementary Material 1 [file 41598_2025_17313_MOESM1_ESM.zip › thermal_images/thermal_frame_0020.png]

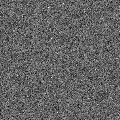

Supplement: Supplementary file 1 — Supplementary Material 1 [file 41598_2025_17313_MOESM1_ESM.zip › thermal_images/thermal_frame_0021.png]

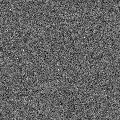

Supplement: Supplementary file 1 — Supplementary Material 1 [file 41598_2025_17313_MOESM1_ESM.zip › thermal_images/thermal_frame_0022.png]

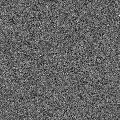

Supplement: Supplementary file 1 — Supplementary Material 1 [file 41598_2025_17313_MOESM1_ESM.zip › thermal_images/thermal_frame_0023.png]

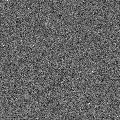

Supplement: Supplementary file 1 — Supplementary Material 1 [file 41598_2025_17313_MOESM1_ESM.zip › thermal_images/thermal_frame_0024.png]

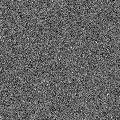

Supplement: Supplementary file 1 — Supplementary Material 1 [file 41598_2025_17313_MOESM1_ESM.zip › thermal_images/thermal_frame_0025.png]

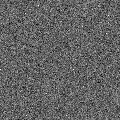

Supplement: Supplementary file 1 — Supplementary Material 1 [file 41598_2025_17313_MOESM1_ESM.zip › thermal_images/thermal_frame_0026.png]

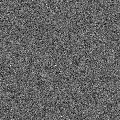

Supplement: Supplementary file 1 — Supplementary Material 1 [file 41598_2025_17313_MOESM1_ESM.zip › thermal_images/thermal_frame_0027.png]

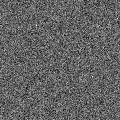

Supplement: Supplementary file 1 — Supplementary Material 1 [file 41598_2025_17313_MOESM1_ESM.zip › thermal_images/thermal_frame_0028.png]

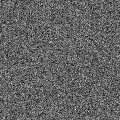

Supplement: Supplementary file 1 — Supplementary Material 1 [file 41598_2025_17313_MOESM1_ESM.zip › thermal_images/thermal_frame_0029.png]

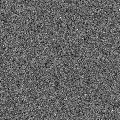

Supplement: Supplementary file 1 — Supplementary Material 1 [file 41598_2025_17313_MOESM1_ESM.zip › thermal_images/thermal_frame_0030.png]

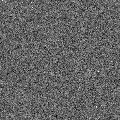

Supplement: Supplementary file 1 — Supplementary Material 1 [file 41598_2025_17313_MOESM1_ESM.zip › thermal_images/thermal_frame_0031.png]

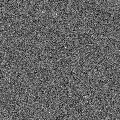

Supplement: Supplementary file 1 — Supplementary Material 1 [file 41598_2025_17313_MOESM1_ESM.zip › thermal_images/thermal_frame_0032.png]

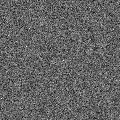

Supplement: Supplementary file 1 — Supplementary Material 1 [file 41598_2025_17313_MOESM1_ESM.zip › thermal_images/thermal_frame_0033.png]

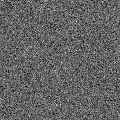

Supplement: Supplementary file 1 — Supplementary Material 1 [file 41598_2025_17313_MOESM1_ESM.zip › thermal_images/thermal_frame_0034.png]

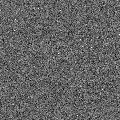

Supplement: Supplementary file 1 — Supplementary Material 1 [file 41598_2025_17313_MOESM1_ESM.zip › thermal_images/thermal_frame_0035.png]

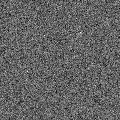

Supplement: Supplementary file 1 — Supplementary Material 1 [file 41598_2025_17313_MOESM1_ESM.zip › thermal_images/thermal_frame_0036.png]

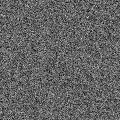

Supplement: Supplementary file 1 — Supplementary Material 1 [file 41598_2025_17313_MOESM1_ESM.zip › thermal_images/thermal_frame_0037.png]

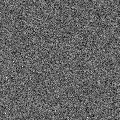

Supplement: Supplementary file 1 — Supplementary Material 1 [file 41598_2025_17313_MOESM1_ESM.zip › thermal_images/thermal_frame_0038.png]

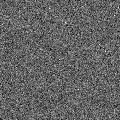

Supplement: Supplementary file 1 — Supplementary Material 1 [file 41598_2025_17313_MOESM1_ESM.zip › thermal_images/thermal_frame_0039.png]

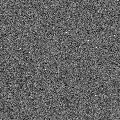

Supplement: Supplementary file 1 — Supplementary Material 1 [file 41598_2025_17313_MOESM1_ESM.zip › thermal_images/thermal_frame_0040.png]

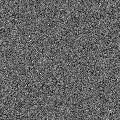

Supplement: Supplementary file 1 — Supplementary Material 1 [file 41598_2025_17313_MOESM1_ESM.zip › thermal_images/thermal_frame_0041.png]

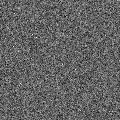

Supplement: Supplementary file 1 — Supplementary Material 1 [file 41598_2025_17313_MOESM1_ESM.zip › thermal_images/thermal_frame_0042.png]

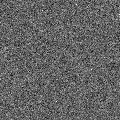

Supplement: Supplementary file 1 — Supplementary Material 1 [file 41598_2025_17313_MOESM1_ESM.zip › thermal_images/thermal_frame_0043.png]

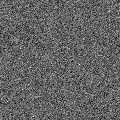

Supplement: Supplementary file 1 — Supplementary Material 1 [file 41598_2025_17313_MOESM1_ESM.zip › thermal_images/thermal_frame_0044.png]

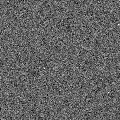

Supplement: Supplementary file 1 — Supplementary Material 1 [file 41598_2025_17313_MOESM1_ESM.zip › thermal_images/thermal_frame_0045.png]

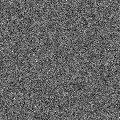

Supplement: Supplementary file 1 — Supplementary Material 1 [file 41598_2025_17313_MOESM1_ESM.zip › thermal_images/thermal_frame_0046.png]

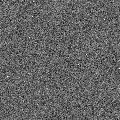

Supplement: Supplementary file 1 — Supplementary Material 1 [file 41598_2025_17313_MOESM1_ESM.zip › thermal_images/thermal_frame_0047.png]

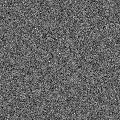

Supplement: Supplementary file 1 — Supplementary Material 1 [file 41598_2025_17313_MOESM1_ESM.zip › thermal_images/thermal_frame_0048.png]

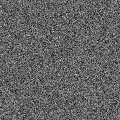

Supplement: Supplementary file 1 — Supplementary Material 1 [file 41598_2025_17313_MOESM1_ESM.zip › thermal_images/thermal_frame_0049.png]

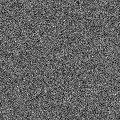

Supplement: Supplementary file 1 — Supplementary Material 1 [file 41598_2025_17313_MOESM1_ESM.zip › thermal_images/thermal_frame_0050.png]

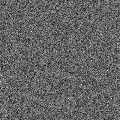

Supplement: Supplementary file 1 — Supplementary Material 1 [file 41598_2025_17313_MOESM1_ESM.zip › thermal_images/thermal_frame_0051.png]

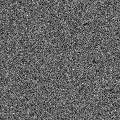

Supplement: Supplementary file 1 — Supplementary Material 1 [file 41598_2025_17313_MOESM1_ESM.zip › thermal_images/thermal_frame_0052.png]

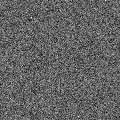

Supplement: Supplementary file 1 — Supplementary Material 1 [file 41598_2025_17313_MOESM1_ESM.zip › thermal_images/thermal_frame_0053.png]

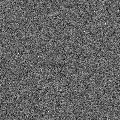

Supplement: Supplementary file 1 — Supplementary Material 1 [file 41598_2025_17313_MOESM1_ESM.zip › thermal_images/thermal_frame_0054.png]

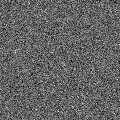

Supplement: Supplementary file 1 — Supplementary Material 1 [file 41598_2025_17313_MOESM1_ESM.zip › thermal_images/thermal_frame_0055.png]

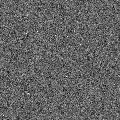

Supplement: Supplementary file 1 — Supplementary Material 1 [file 41598_2025_17313_MOESM1_ESM.zip › thermal_images/thermal_frame_0056.png]

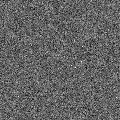

Supplement: Supplementary file 1 — Supplementary Material 1 [file 41598_2025_17313_MOESM1_ESM.zip › thermal_images/thermal_frame_0057.png]

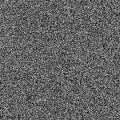

Supplement: Supplementary file 1 — Supplementary Material 1 [file 41598_2025_17313_MOESM1_ESM.zip › thermal_images/thermal_frame_0058.png]

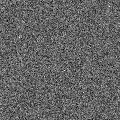

Supplement: Supplementary file 1 — Supplementary Material 1 [file 41598_2025_17313_MOESM1_ESM.zip › thermal_images/thermal_frame_0059.png]

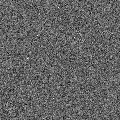

Supplement: Supplementary file 1 — Supplementary Material 1 [file 41598_2025_17313_MOESM1_ESM.zip › thermal_images/thermal_frame_0060.png]

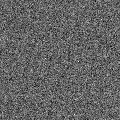

Supplement: Supplementary file 1 — Supplementary Material 1 [file 41598_2025_17313_MOESM1_ESM.zip › thermal_images/thermal_frame_0061.png]

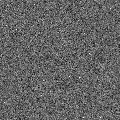

Supplement: Supplementary file 1 — Supplementary Material 1 [file 41598_2025_17313_MOESM1_ESM.zip › thermal_images/thermal_frame_0062.png]

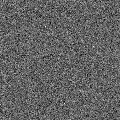

Supplement: Supplementary file 1 — Supplementary Material 1 [file 41598_2025_17313_MOESM1_ESM.zip › thermal_images/thermal_frame_0063.png]

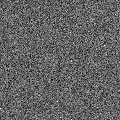

Supplement: Supplementary file 1 — Supplementary Material 1 [file 41598_2025_17313_MOESM1_ESM.zip › thermal_images/thermal_frame_0064.png]

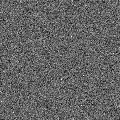

Supplement: Supplementary file 1 — Supplementary Material 1 [file 41598_2025_17313_MOESM1_ESM.zip › thermal_images/thermal_frame_0065.png]

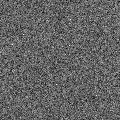

Supplement: Supplementary file 1 — Supplementary Material 1 [file 41598_2025_17313_MOESM1_ESM.zip › thermal_images/thermal_frame_0066.png]

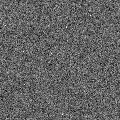

Supplement: Supplementary file 1 — Supplementary Material 1 [file 41598_2025_17313_MOESM1_ESM.zip › thermal_images/thermal_frame_0067.png]

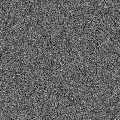

Supplement: Supplementary file 1 — Supplementary Material 1 [file 41598_2025_17313_MOESM1_ESM.zip › thermal_images/thermal_frame_0068.png]

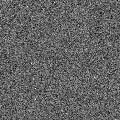

Supplement: Supplementary file 1 — Supplementary Material 1 [file 41598_2025_17313_MOESM1_ESM.zip › thermal_images/thermal_frame_0069.png]

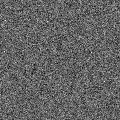

Supplement: Supplementary file 1 — Supplementary Material 1 [file 41598_2025_17313_MOESM1_ESM.zip › thermal_images/thermal_frame_0070.png]

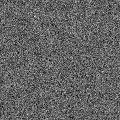

Supplement: Supplementary file 1 — Supplementary Material 1 [file 41598_2025_17313_MOESM1_ESM.zip › thermal_images/thermal_frame_0071.png]

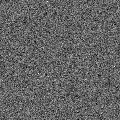

Supplement: Supplementary file 1 — Supplementary Material 1 [file 41598_2025_17313_MOESM1_ESM.zip › thermal_images/thermal_frame_0072.png]

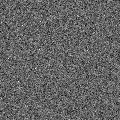

Supplement: Supplementary file 1 — Supplementary Material 1 [file 41598_2025_17313_MOESM1_ESM.zip › thermal_images/thermal_frame_0073.png]

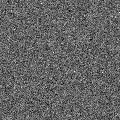

Supplement: Supplementary file 1 — Supplementary Material 1 [file 41598_2025_17313_MOESM1_ESM.zip › thermal_images/thermal_frame_0074.png]

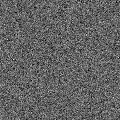

Supplement: Supplementary file 1 — Supplementary Material 1 [file 41598_2025_17313_MOESM1_ESM.zip › thermal_images/thermal_frame_0075.png]

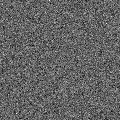

Supplement: Supplementary file 1 — Supplementary Material 1 [file 41598_2025_17313_MOESM1_ESM.zip › thermal_images/thermal_frame_0076.png]

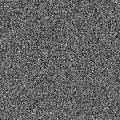

Supplement: Supplementary file 1 — Supplementary Material 1 [file 41598_2025_17313_MOESM1_ESM.zip › thermal_images/thermal_frame_0077.png]

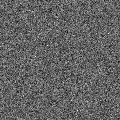

Supplement: Supplementary file 1 — Supplementary Material 1 [file 41598_2025_17313_MOESM1_ESM.zip › thermal_images/thermal_frame_0078.png]

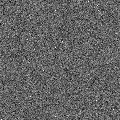

Supplement: Supplementary file 1 — Supplementary Material 1 [file 41598_2025_17313_MOESM1_ESM.zip › thermal_images/thermal_frame_0079.png]

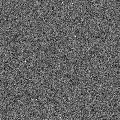

Supplement: Supplementary file 1 — Supplementary Material 1 [file 41598_2025_17313_MOESM1_ESM.zip › thermal_images/thermal_frame_0080.png]

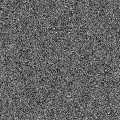

Supplement: Supplementary file 1 — Supplementary Material 1 [file 41598_2025_17313_MOESM1_ESM.zip › thermal_images/thermal_frame_0081.png]

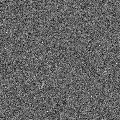

Supplement: Supplementary file 1 — Supplementary Material 1 [file 41598_2025_17313_MOESM1_ESM.zip › thermal_images/thermal_frame_0082.png]

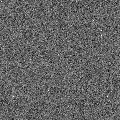

Supplement: Supplementary file 1 — Supplementary Material 1 [file 41598_2025_17313_MOESM1_ESM.zip › thermal_images/thermal_frame_0083.png]

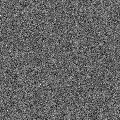

Supplement: Supplementary file 1 — Supplementary Material 1 [file 41598_2025_17313_MOESM1_ESM.zip › thermal_images/thermal_frame_0084.png]

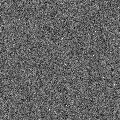

Supplement: Supplementary file 1 — Supplementary Material 1 [file 41598_2025_17313_MOESM1_ESM.zip › thermal_images/thermal_frame_0085.png]

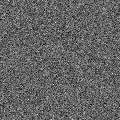

Supplement: Supplementary file 1 — Supplementary Material 1 [file 41598_2025_17313_MOESM1_ESM.zip › thermal_images/thermal_frame_0086.png]

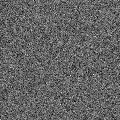

Supplement: Supplementary file 1 — Supplementary Material 1 [file 41598_2025_17313_MOESM1_ESM.zip › thermal_images/thermal_frame_0087.png]

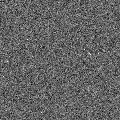

Supplement: Supplementary file 1 — Supplementary Material 1 [file 41598_2025_17313_MOESM1_ESM.zip › thermal_images/thermal_frame_0088.png]

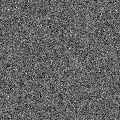

Supplement: Supplementary file 1 — Supplementary Material 1 [file 41598_2025_17313_MOESM1_ESM.zip › thermal_images/thermal_frame_0089.png]

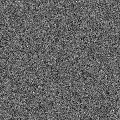

Supplement: Supplementary file 1 — Supplementary Material 1 [file 41598_2025_17313_MOESM1_ESM.zip › thermal_images/thermal_frame_0090.png]

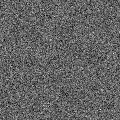

Supplement: Supplementary file 1 — Supplementary Material 1 [file 41598_2025_17313_MOESM1_ESM.zip › thermal_images/thermal_frame_0091.png]

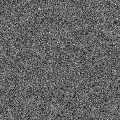

Supplement: Supplementary file 1 — Supplementary Material 1 [file 41598_2025_17313_MOESM1_ESM.zip › thermal_images/thermal_frame_0092.png]

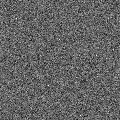

Supplement: Supplementary file 1 — Supplementary Material 1 [file 41598_2025_17313_MOESM1_ESM.zip › thermal_images/thermal_frame_0093.png]

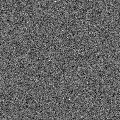

Supplement: Supplementary file 1 — Supplementary Material 1 [file 41598_2025_17313_MOESM1_ESM.zip › thermal_images/thermal_frame_0094.png]

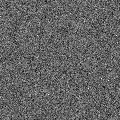

Supplement: Supplementary file 1 — Supplementary Material 1 [file 41598_2025_17313_MOESM1_ESM.zip › thermal_images/thermal_frame_0095.png]

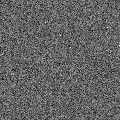

Supplement: Supplementary file 1 — Supplementary Material 1 [file 41598_2025_17313_MOESM1_ESM.zip › thermal_images/thermal_frame_0096.png]

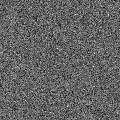

Supplement: Supplementary file 1 — Supplementary Material 1 [file 41598_2025_17313_MOESM1_ESM.zip › thermal_images/thermal_frame_0097.png]

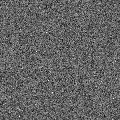

Supplement: Supplementary file 1 — Supplementary Material 1 [file 41598_2025_17313_MOESM1_ESM.zip › thermal_images/thermal_frame_0098.png]

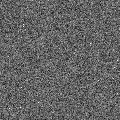

Supplement: Supplementary file 1 — Supplementary Material 1 [file 41598_2025_17313_MOESM1_ESM.zip › thermal_images/thermal_frame_0099.png]

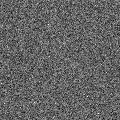

Supplement: Supplementary file 1 — Supplementary Material 1 [file 41598_2025_17313_MOESM1_ESM.zip › thermal_images/thermal_frame_0100.png]
